# Human Interleukin-34 facilitates microglia-like cell differentiation and persistent HIV-1 infection in humanized mice

**DOI:** 10.1186/s13024-019-0311-y

**Published:** 2019-03-05

**Authors:** Saumi Mathews, Amanda Branch Woods, Ikumi Katano, Edward Makarov, Midhun B. Thomas, Howard E. Gendelman, Larisa Y. Poluektova, Mamoru Ito, Santhi Gorantla

**Affiliations:** 1 Department of Pharmacology and Experimental Neuroscience, University of Nebraska Medical Center, 985880 Nebraska Medical Center, Omaha, NE 68198-5880 USA; 2 Central Institute for Experimental Animals, Kawasaki-ku, Kawasaki, Japan

**Keywords:** Microglia, Hematopoietic stem cells, Humanized mice, HIV-1 infection

## Abstract

**Background:**

Microglia are the principal innate immune defense cells of the centeral nervous system (CNS) and the target of the human immunodeficiency virus type one (HIV-1). A complete understanding of human microglial biology and function requires the cell’s presence in a brain microenvironment. Lack of relevant animal models thus far has also precluded studies of HIV-1 infection. Productive viral infection in brain occurs only in human myeloid linage microglia and perivascular macrophages and requires cells present throughout the brain. Once infected, however, microglia become immune competent serving as sources of cellular neurotoxic factors leading to disrupted brain homeostasis and neurodegeneration.

**Methods:**

Herein, we created a humanized bone-marrow chimera producing human “microglia like” cells in NOD.Cg-*Prkdc*^*scid*^*Il2rg*^*tm1Sug*^Tg(CMV-IL34)1/Jic mice. Newborn mice were engrafted intrahepatically with umbilical cord blood derived CD34+ hematopoietic stem progenitor cells (HSPC). After 3 months of stable engraftment, animals were infected with HIV-1_ADA_, a myeloid-specific tropic viral isolate. Virologic, immune and brain immunohistology were performed on blood, peripheral lymphoid tissues, and brain.

**Results:**

Human interleukin-34 under the control of the cytomegalovirus promoter inserted in NSG mouse strain drove brain reconstitution of HSPC derived peripheral macrophages into microglial-like cells. These human cells expressed canonical human microglial cell markers that included CD14, CD68, CD163, CD11b, ITGB2, CX3CR1, CSFR1, TREM2 and P2RY12. Prior restriction to HIV-1 infection in the rodent brain rested on an inability to reconstitute human microglia. Thus, the natural emergence of these cells from ingressed peripheral macrophages to the brain could allow, for the first time, the study of a CNS viral reservoir. To this end we monitored HIV-1 infection in a rodent brain. Viral RNA and HIV-1p24 antigens were readily observed in infected brain tissues. Deep RNA sequencing of these infected mice and differential expression analysis revealed human-specific molecular signatures representative of antiviral and neuroinflammatory responses.

**Conclusions:**

This humanized microglia mouse reflected human HIV-1 infection in its known principal reservoir and showed the development of disease-specific innate immune inflammatory and neurotoxic responses mirroring what can occur in an infected human brain.

**Electronic supplementary material:**

The online version of this article (10.1186/s13024-019-0311-y) contains supplementary material, which is available to authorized users.

## Background

Human immunodeficiency virus-1 (HIV-1) invades the central nervous system (CNS) early after viral infection [1]. Virus persists in brain despite avid host antiviral cellular and humoral immune responses and during combination antiretroviral therapy (cART) [2]. Low-level viral persistence is associated with neurocognitive dysfunction which is commonplace despite cART. Disease continues to affect up to one third or more of all infected patients [3]. Studies of viral pathogenesis, therapies and the natural history of brain infection remain incomplete based on the available animal models which reflect human disease. There are few ways to replicate virus-induced innate immune responses as they affect neural signaling and neurodegeneration, mostly using in vitro model systems. Indeed, a pressing need remains to develop human disease models of HIV brain infection. This, however, requires the presence of relevant human perivascular macrophages and microglia. Without such cells the ability to represent viral restriction and latency in the nervous system during cART is limited. Humanized mice developed by reconstituting immune compromised mice with human hematolymphoid system have been important tools to study hematopoiesis, cancer, autoimmunity, infections and degenerative disorders [4–6]. Previously we used humanized mice transplanted with human hematopietic stem cells (HSPC) in NOD/Scid IL2Rγ−/− (NSG/NOG) mice to study HIV persistence in brain and its associated neurodegenration [7–10]. However the model proved imperfect and many aspects of human brain infection could not be recapitulated. This was due, in large measure, to the fact that such mice do not have the abilities to reconstitute human microglia following human HSPC transplantation. Human monocyte-macrophages were found nearly exclusively in meningeal and perivascular areas and very rare are few microglia-like cells located in the parenchyma of the mouse brain [8, 11]. Moreover, no alterations in such cell ontogeny or robust differentiation of macrophages to microglia were observed even following inflammatory or infectious diseases including HIV-1. Notably, such failures present a critical barrier in progressing studies of HIV-1 brain reservoirs and cure strategies. Nonetheless, the abilities to repopulate a murine brain with functional human microglial-like cells could provide an enormous advantage to study both inflammatory and infectious diseases.

We now posit that the lack of human microglial cells in HSPC reconstituted humanized mice could be due to deficits in species-specific cytokine support. While some cytokines-ligand interactions are cross-reactive between human and mouse, certain interactions are either asymmetric or highly species-specific [12]. Those factors play an important role in macrophage-microglial differentiation are the colony stimulating factor-1 (CSF-1) and interleukin 34 (IL-34) [13]. IL-34 has no sequence similarity with CSF-1 [14]. Although both are the ligands for CSF1 receptor (CSF1R), differentiation of bone marrow (BM)-derived progenitor cells from monocytes to tissue macrophages and dendritic cells require CSF-1. IL-34, in contrast is required specifically for the development of microglia and Langerhans cells [15–17]. The existing immunodeficient transgenic mouse strains that express human CSF-1 [18, 19] do not support human microglial development following human HSPC reconstitution (our unpublished observation). Further, multiple mouse strains expressing human CSF-1 including transgenic or knock-in have not reported spontaneous development of microglia [18–21]. To these ends, we developed a human IL-34 (hIL-34) transgenic NOG mice that successfully induced the development of human microglia-like cells seen following human HSPC reconstitution. These microglial-like cells expressed all major myeloid/ microglial cell markers cluster differentiation (CD) 14, CD68, CD163, CD11b, integrin beta 2 (ITGB2), C-X3-C Motif Chemokine Receptor 1 (CX3CR1), CSFR1, triggering Receptor Expressed On Myeloid Cells 2 (Trem2) and Purinergic Receptor P2Y (P2Ry12), and were readily infected by HIV-1. Infection induced human-cell specific molecular events linked to antiviral defense, immune activation and neuroinflammation and reflective to what was previously reported in an infected human host [22–24].

## Methods

### Ethics

Animal procedures strictly followed the Institutional Animal Care and Use Committee guidelines approved protocols at University of Nebraska Medical Center (UNMC) (IACUC 18–109) and Institutional Guidelines (11004) approved by the Animal Experimentation Committee of Central Institute of Experimental Animals (CIEA).

### Generation of NOG-hIL-34 mice

NOG (formally, NOD.*Cg-Prkdc*^*scid*^*il2g*^*tmlSug*^/Jic) and NOD/ShiJcl (NOD) were used. NOG mice were maintained in the CIEA under specific-pathogen-free conditions. NOD mice were purchased from CIEA (Tokyo, Japan). To generate the hIL-34 expressing transgenic NOG mouse (NOG-hIL-34), a linearized DNA vector (pCMV6-XL4) containing human IL-34 cDNA (Origene Technologies, Inc., Rockville, MD, USA) under the control of a CMV promoter, was microinjected into fertilized eggs obtained by mating NOG and NOD mice. Out of 26 weaning pups obtained, 3 (#11, #13 and #24) were positive by polymerase chain reactions (PCR) multiplying hIL-34 cDNA. The positive pups were further backcrossed to NOG mice to establish NOG-hIL-34 mice. Out of the three lines generated, the line that is highest for the peripheral blood human IL-34 (#11 with 550 pg/ml) was selected to expand the colony. Mice are currently maintained as heterozygous for human IL-34. We did not observe any dectectable abnormalities with growth, shape, size and normal activity. The weight of the mice ranged from 16 to 20 g. Females were smaller than males. The litter size ranged from 5 to 12 pups. They can live over 1.5 years of age without any health issues. Targeted locus amplification (TLA) analyses by Cergentis, Utrecht, Netherlands, was performed to identify the transgene (TG): CMV-XL4-hIL-34 sequence integrated sites in the mouse genome, break point sequences between TG and genome, genetic variations of the TG and to assess the structural variants around the transgene. Bone marrow cells were used and processed for TLA and sequencing [25], using two primer sets 1. ACTAATGACCCCGTAATTGA and GTCAATGACGGTAAATGGC, and 2. TTTGTGATGCTCGTCAGG and GCAGATTACGCGCAGAAA.

### Human IL-34 quantitation by ELISA

Transgenic expression of hIL-34 was evaluated by quantifying hIL-34 in mouse plasma (1:10 dilution) from NOG-hIL-34 (*n* = 6). NOG control (*n* = 5) were included in the assay to serve as negative controls. hIL-34 was quantified using human IL-34 ELISA Quantitation set (R&D systems, MN, USA) as per the manufacturer’s instructions. Absorbance was read at 450 nm on a SpectraMax M3 (Molecular Devices, USA). The hIL-34 ELISA kit sensitivity was low with high background. 1:10 dilution of plasma was used for the assay. Some samples were excluded because of lack of detection due to higher dilution used with sample availability.

### RT-PCR for hIL-34 transcripts

RNA from spleen, lung, liver, kidney, skin and brain tissues was obtained by homogenizing in Trizol solution using a Qiagen Tissue Lyzer II (Valencia, CA, USA) and isolating RNA by phenol-chloroform method. cDNA was synthesized from RNA using Verso cDNA Synthesis Kit (Thermo Scientific, Vilnius, Lithuania) as per manufacturer’s instructions. Real-time PCR amplification was performed on the ABI Step One Plus machine (Applied Biosystems, MA, USA) using TaqMan detection chemistry. We compared the expression of hIL-34 (Hs01050926_m1) in CD34-NOG-hIL-34 mouse samples against NOG mouse samples. Mouse IL-34 was also assayed using Mm01243248_m1 primer/probe set. GAPDH (Mm99999915_g1) was used as housekeeping gene. The real-time PCR settings were as follows: 50 °C for 2 min, 95 °C for 10 min, 40 cycles of 95 °C for 15 s, and 60 °C for 1 min. The fold change of the target gene expression relative to GAPDH was determined in the transgenic group and the control group. This was performed by using threshold cycle (C_T_) and the 2^−ΔΔCT^ method, where ΔC_T_ = C_T_IL34–C_T_GAPDH and ΔΔC_T_ = ΔC_T_transgenic–ΔC_T_control. Human IL-34 expression was analyzed in tissues from 17 NOG-hIL-34 mice, except for skin (*n* = 5), and compare with NOG controls (n = 5).

### Human CD34+ HSPC isolation

Human CD34+ HSPC were obtained from human cord blood of healthy full-term newborns after getting parental written informed consent and approval from Institutional Review Board of UNMC (Department of Gynecology and Obstetrics). After density gradient centrifugation of cord blood in leukocyte separation medium (MP Biomedicals, Santa Ana, CA, USA) at 300 g for 35 min, the buffy coat was collected for enriching CD34^+^ cells using immunomagnetic beads according to the manufacturer’s instructions (CD34^+^ selection kit; Miltenyi Biotec Inc., Auburn, CA). Purity of isolated CD34^+^ cells was evaluated by flow cytometry. CD34^+^ HSPCs were either used fresh or stored in liquid nitrogen using freezing medium with 50% Bovine serum albumin (Sigma-Aldrich, St Louis, MO, USA), 40% Iscove’smodified Dulbeccos’s medium (GIBCO, Life technologies, Carlsbad, CA, USA) and 10% dimethyl sulfoxide (DMSO; Sigma-Aldrich, St Louis, MO, USA).

### Human CD34^+^ HSPC transplantation

NOG-hIL-34 mice were bred and housed in the pathogen-free facility at the UNMC. New born pups (post-natal day 0–1) were irradiated with 1 Gy (RS 2000 X-ray Irradiator, Rad Source Technologies, Inc., Suwanee, GA, USA). After 4 h of irradiation, pups were intrahepatically injected with 10^5^ CD34^+^ HSPCs. A total of 17 reconstituted animals were used in the study. For comparison, NSG (NOD.Cg-*Prkdc*^*scid*^ *Il2rg*^*tm1Wjl*^/SzJ, Stock no.:005557 from Jackson laboratories) mice were also reconstituted with CD34^+^ HSPCs. Engraftment of human leukocytes were examined by flow cytometry analysis of blood samples collected from the facial vein after 12 weeks post-engraftment.

### Flow cytometry

Blood samples were collected from a facial vein or by direct heart puncture after euthanasia in ethylene diamine tetraacetic acid (EDTA)-containing tubes (BD Microtainer, Franklin Lakes, NJ, USA) and centrifuged at 1800 rpm for 8 min. Splenocytes were collected by crushing the spleen tissue through 40 μ cell strainer. Blood cells and splenocytes were reconstituted in a fluorescence activated cell sorting (FACS) buffer (2% FBS in phosphate-buffered saline) and incubated with a cocktail of antibodies against human immune cell markers, CD45+ fluorescein isothiocyanate (FITC), CD3+ Alexa Fluor 700 (AF700), CD19+ Phycoerythrin-Cyanin 5 (PE-Cy5), CD4+ allophycocyanin (APC), CD8+ Brilliant Violet 421 (BV421) and CD14+ Phycoerythrin (PE), for 30 min at 4 °C. All antibodies and isotype controls were obtained from BD Biosciences, USA. Red blood cells were lysed by FACS lysing solution (BD Biosciences, USA). Stained cells were washed with FACS buffer and fixed with 2% paraformaldehyde. Data acquisition was carried out with acquisition software FACS Diva v6 (BD Biosciences, USA) in a BD LSR2 flow cytometer, and data were analyzed using FLOWJO analysis software v10.2 (Tree Star, USA). Gates were assigned according to the appropriate control population.

### HIV-1 infection

Mice with established human hemato-lymphoid reconstitution (∼6–8 months of age) and positive for IL-34 expression were intraperitoneally infected (*n* = 12) with the 10^4^ tissue culture infectious dose_50_ (TCID_50_) of macrophage-tropic HIV-1_ADA_ strain. Viral stocks were prepared as described previously [26]. Mice were bled at 3 weeks post infection and euthanized 6 weeks after the infection to collect blood and tissues for analyses.

### Measurements of HIV-1 in plasma, spleen and brain

Viral RNA copies in the mouse plasma, isolated from blood either collected from facial vein bleeding or endpoint cardiocentesis, were determined by using a COBAS Amplicor System v1.5 kit (Roche Molecular Diagnostics, Pleasanton, CA, USA) three and 6 weeks after post infection. Expression of HIV-1 group-specific antigen (gag) RNA in brain were analyzed as described above on the ABI Step One Plus real-time PCR machine (Applied Biosystems, MA, USA) using TaqMan detection chemistry. The primers and probe used for the PCR were: antisense 5′-ATCTGGCCTGGTGCAATAGG-3′, sense 5′-ACATCAAGCAGCCATGCAAAAT-3′ (Invitrogen, Life technologies, Pittsburgh, PA, USA) and TaqMan probe FAM-CATCAATGAGGAAGCTGCAGAATGGGATAGA-TAMRA (Applied Biosystems, Foster City, CA, USA). Log fold change expression was calculated using ΔΔC_T_ method after normalization with an endogenous mouse GAPDH (Mm99999915_g1) transcript expression of total RNA.

### Immunohistochemistry

Tissues (spleen, liver, and left hemisphere of brain) were fixed with 4% paraformaldehyde for 24 h at room temperature, and then embedded in paraffin. Antigen retrieval of paraffin-embedded 5-μm thick tissue sections were performed with Declere/ trilogy Solution (Sigma-Aldrich, St Louis, MO, USA) according to the manufacturer’s instructions. Immunohistochemistry was performed using EXPOSE Mouse and Rabbit Specific horse radish peroxidase (HRP)/ 3,3′-Diaminobenzidine (DAB) Detection IHC Kit (Abcam, Cambridge, MA, USA) as per manufacturer’s instructions. Primary antibodies used were HLA-DQ/DR/DP (CR3/43) (1:100; Novus Biologicals, Littleton, CO, USA), CD14 (EPR3653) (1:500; Abcam, Cambridge, MA, USA), CD68 (KP1) (1:100; Dako, Carpenteria, CA, USA,) CD163 (10D6) (1:100; Thermoscientific, Rockford, IL, USA), P2Ry12 (1:200; Sigma life science, Inc. ST Louis, MO, USA), Iba1 (1:500; Wako life sciences, Richmond, VA, USA) and HIV-1p24 (Kal1) (1:20; Dako, Carpenteria, CA, USA). The nuclei were counterstained with Mayer’s hematoxylin. Non-humanized mouse tissues were included in every staining protocol as negative controls for testing the specificity of the antibodies to human proteins. Bright field images were captured and photographed using 20× and 40× objectives on a Nuance Multispectral Tissue Imaging system (CRi, Wobum, MA). For quantification, HLA-DR stained sections were scanned using a high-resolution scanner (Ventana Medical Systems, Inc., Oro Valley, AZ, USA). DEFINIENS Tissue Studio® software (Definiens AG, Munich, Germany) was used to analyze the brain sections stained for HLA-DR.

### RNAScope®

RNAScope® (Advanced Cell Diagnostics, Hayward, CA) was performed for the detection of human IL-34 on paraffin embedded sections as per manufacturer’s instructions. Briefly, 5 μm thick de-paraffinized and dehydrated formalin-fixed paraffin-embedded brain sections were pretreated with hydrogen peroxide at room temperature for 10 min, target retrieval solution for 8 min at 100 °C and then protease IV at 40 °C for 15 min in a HybEZ hybridization oven. Hybridization with target probe, pre-amplification, amplification, and chromogenic detection using DAB was carried out as per manufacturer’s instructions in HybEZ oven at 40 °C.

For IL-34 RNA detection, a channel 1 anti-sense Hs-IL34-No-XMm, which contains 20 probe pairs targeting 38–1774 of hIL-34 was used in the single-plex chromogenic assay. For morphological detection of HIV RNA copies in spleen and brain tissues, a channel 1 anti-sense HIV-1 Clade B target probe, which contains 78 probe pairs targeting base pairs 854–8291 of HIV-1 was used. Positive expression was indicated by the presence of brown dots in the infected cells.

### Immunofluorescence

Paraffin embedded five-micron sections were processed and blocked with 10% normal goat serum with 0.5% tween in 1X tris buffered saline. Primary antibodies used in the study were mouse (Ms) anti-human HLA- DQ/DR/DP(CR3/43) (1:100; Novus Biologicals, Littleton, CO, USA), Ms. anti-HIV-1p24 (Kal1) (1:20; Dako, Carpenteria, CA, USA), anti-synaptophysin (YE269) (1:800; Abcam, Cambridge, MA, USA), rabbit (Rb) anti-MAP2 (1:500; Millipore, Burlington, MA, USA), Rb anti-Neurofilament H (1:400; Millipore, Burlington, MA, USA), Polyclonal Rb anti-glial fibrillary acidic protein (1:1000; Dako, Carpenteria, CA, USA) and Rb anti-Iba1 (1:500; Wako life sciences, Richmond, VA, USA). Secondary antibodies were Alexa Fluor 488-conjugated goat anti-Rb IgG (1:200; Invitrogen, Grand Island, NY, USA) and Alexa Fluor 594- conjugated goat anti-ms IgG (1:200; Invitrogen). A Zeiss LSM710 confocal system (Carl Zeiss Microscopy, Jena, Germany) were used for immunofluorescent imaging and images were taken at 63x.

For quantification of the number of human microglial cells, sagittal sections from each mouse (*n* = 3) were double immunostained for Iba1 and HLA-DR. A minimum of 2–4 selected field of views of the same brain region under 400× magnification were counted for HLA-DR+/Iba1 + (human microglia) and Iba1+ (mouse microglia) using Nuance Multispectral Tissue Imaging system (CRi, Wobum, MA).

### Next generation sequencing

Brain tissues (four uninfected CD34-NOG-hIL-34, four HIV-1-infected CD34-NOG-hIL-34 and four NOG controls) were flash frozen in liquid nitrogen and stored in − 80 °C. Tissue RNA was isolated using Trizol method as described above. RNA was further subjected for RNA cleanup using RNeasy mini columns and DNase is removed using RNase- free DNase set (Qiagen, CA, USA). Nucleic acid integrity was analyzed and the RNA samples were deep sequenced using 100 bp/read, ≤40 million reads/sample using an Illumina HiSeq 2500 Sequence Analyzer (Illumina, Inc., San Diego, CA, USA). Reads were trimmed using the fqtrim (ccb.jhu.edu/software/fqtrim/index.shtml) software to remove ambiguous bases from the reads. Quality was assessed for each sample with FASTQC before and after trimming. The reads were aligned with STAR-2.5.3a (https://github.com/alexdobin/STAR) to mouse reference genome, GRCm38.p3, (https://uswest.ensembl.org/index.html) with default parameters, and then were quantified by RSEM 1.2.21 (https://deweylab.github.io/RSEM/), using Ensemble annotations (Additional file 1: Figure S9). The gene and transcript abundance were measured as Transcripts Per Kilobase Million (TPM) values. TPM is calculated by normalizing the gene length, followed by the sequencing depth to make easier comparison of the proportion of reads that mapped to a gene in each sample. The unmapped reads for CD34-NOG-hIL-34 samples were further aligned to human reference genome, GrCh37 (https://uswest.ensembl.org/index.html), using the same pipeline and comparison analysis was performed between uninfected and HIV-1infected samples. The reads that didn’t align to human were further aligned to the HIV genome using STAR and quantified by RSEM. Count and expression data were filtered to exclude features aside from protein-coding. This filtered subset of genes was then used to [1] examine the differential expression of various genes between the sample groups in the R statistical software environment with packages from Bioconductor, [2] identify pathways using Ingenuity pathway analysis (https://www.qiagen-bioinformatics.com). We identified differentially expressed genes with TPM values above 2 and *p* values < 0.05. The top ranking upregulated and down regulated genes were selected to plot the graphs. We compared the available literature on genes expressed by microglia and genes differentially expressed in response to HIV infection.

### Statistical analysis

Data was analyzed and plotted using GraphPad prism 7 (Graphpad, USA) and expressed as mean ± standard error mean (SEM). For transcriptome analysis, the data obtained from was expressed as the mean ± standard deviation for each group. Student t-test was performed using R/Bioconductor packages. The Benjamini-Hochberg (BH) adjusted p values were also calculated to adjust for multiple-testing caused false discovery rate (FDR). The *p*-value< 0.05 was considered to indicate a statistically significant difference.

## Results

### Humanized hIL34 transgenic mice

**Fig. 1:**
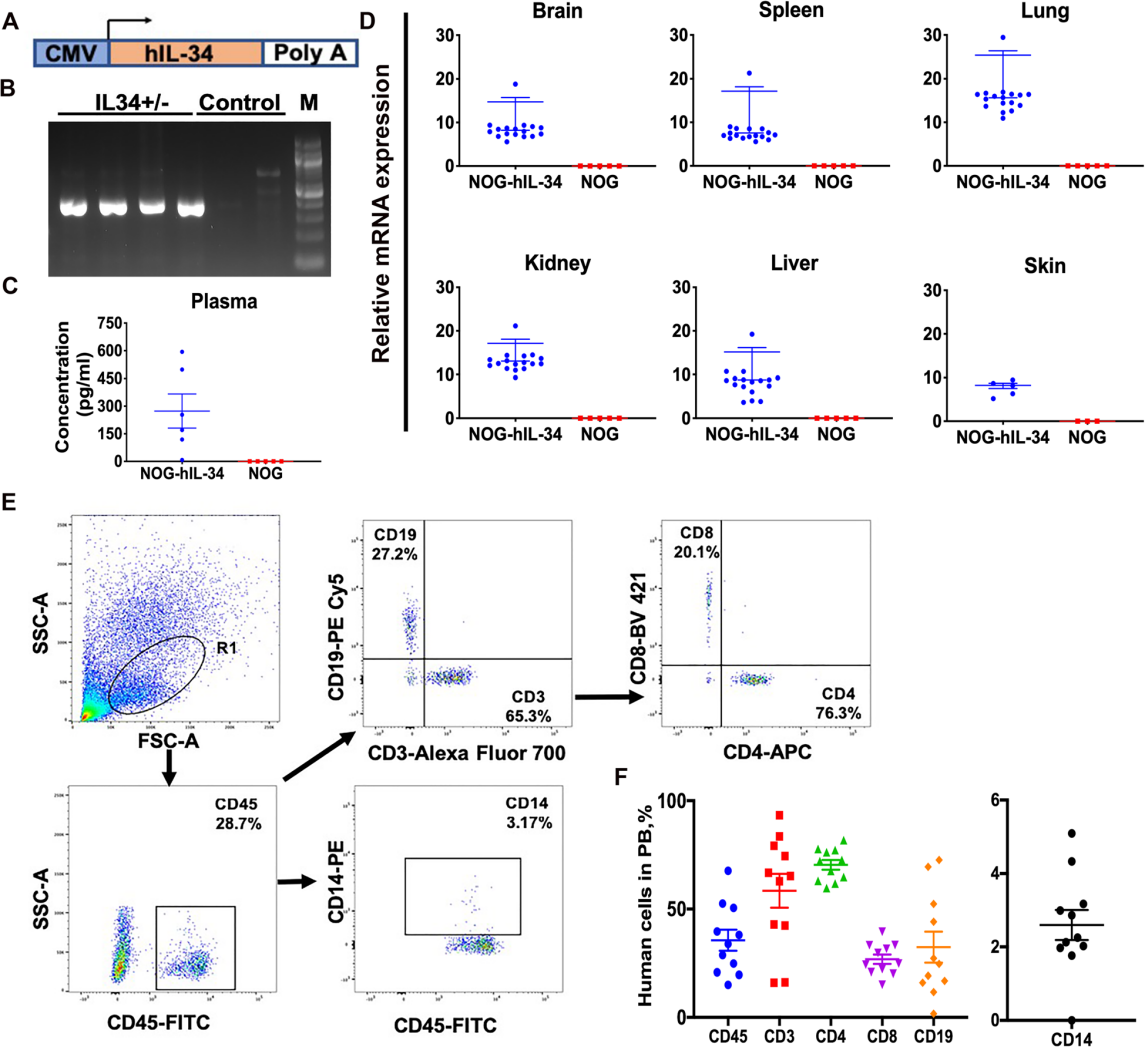
Generation and characterization of NOD.Cg-Prkdc^scid^ Il2rg^tm1Sug^ Tg (CMV-IL34)1/Jic (NOG-hIL-34) mice. **a** NOG-hIL-34 transgenic mice were created in NOD.*Cg-Prkdc*^*scid*^*il2g*^*tmlSug*^/Jic mice by inserting vector containing transgene (Tg), hIL-34, under CMV promoter. **b** NOG-hIL-34 mice were identified by PCR analysis of ear DNA that amplify hIL-34 (358 bp) in homozygous mice. No bands were detected in non-transgenic NOG controls. A representative gel is shown here. Analysis was done for all 17 NOG-hIL-34 being used in the study and confirmed with the presence of hIL-34 genomic DNA. **c** hIL-34 expression in plasma was confirmed by ELISA (NOG-hIL-34, *n* = 6; NOG control, *n* = 5). **d** Tissue specific expression of hIL-34 was observed by real time PCR using total RNA isolated from brain, spleen, lung, kidney, liver and skin of NOG-hIL-34 mice (*n* = 17, except for skin tissue n = 5) compared to NOG controls (n = 5). **e, f** Establishment of human peripheral hematolymphoid system in CD34-NOG-hIL-34 mice. **e** Flow cytometry analysis of peripheral blood at 6 months age and gating strategy Representative plots of human cluster of differentiation (CD) 45 positive cells and human CD3, CD19 and CD14 positive cells from human CD45^+^ gate. **f** Percentage of human cell subtypes in the peripheral blood of CD34-NOG-hIL-34 mice used in the study. Each symbol represents an individual mouse, n = 17

NOD.Cg-*Prkdc*^*scid*^*Il2rg*^*tm1Sug*^Tg(CMV-IL34)1/Jic (NOG-hIL-34), a human IL-34 transgenic mouse model on NOG^CIEA^ background, was created by inserting a vector containing hIL-34 transgene under the CMV promoter (Fig. 1a). NOG-hIL-34 mice were identified by PCR analysis of ear DNA that amplify hIL-34 (358 bp) transcript (Fig. 1b). TLA analyses showed that transgene was integrated in chr1:25,127,822-25,127,823. No structural rearrangements were found in the host genome around the integration site. (Additional file 1: Figure S1). According to the refseq integration was in intronic region of *Adgrb3*. RNAseq analyses revealed no alterations in *Adgrb3* gene expression between NOG and NOG-hIL-34 mice (data not shown). Human IL-34 expression in mouse tissues including brain was confirmed by ELISA, RT-PCR and RNAScope® analyses (Fig. 1c,d) (Additional file 1: Figure S2). Expression of mouse IL-34 in brain were not significantly different between NOG and NOG-hIL-34 mice. Humanization of NOG-hIL-34 mice (CD34-NOG- hIL-34) followed standard methods where human CD34^+^ HSPC are transplanted intrahepatically at birth after conditioning by irradiation [27]. Stable engraftment with human immune system consisting human lymphoid and myeloid cells was achieved in CD34-NOG-hIL-34 mice (Fig. 1e,f), comparable to CD34-NSG (Additional file 1: Figure S3) [28–31]. Such human immune cell reconstitution levels are also similar with other existing humanized mouse models [32]. In CD34-NOG-hIL-34 mice, CD14+ monocyte/macrophages were significantly higher in blood compared to CD34-NSG mice (0.59 ± 0.1 vs 3.1 ± 0.7, *p* < 0.001), however, not as high as in HSPC transplanted human CSF-1, CSF2/IL3 and thrombopoietin transgenic mouse model, where human CD33+ myeloid cells were ~ 60% of circulating human CD45+ cells [19].

### Human microglial-like cells in the hIL-34 trasgenic mouse brain

**Fig. 2:**
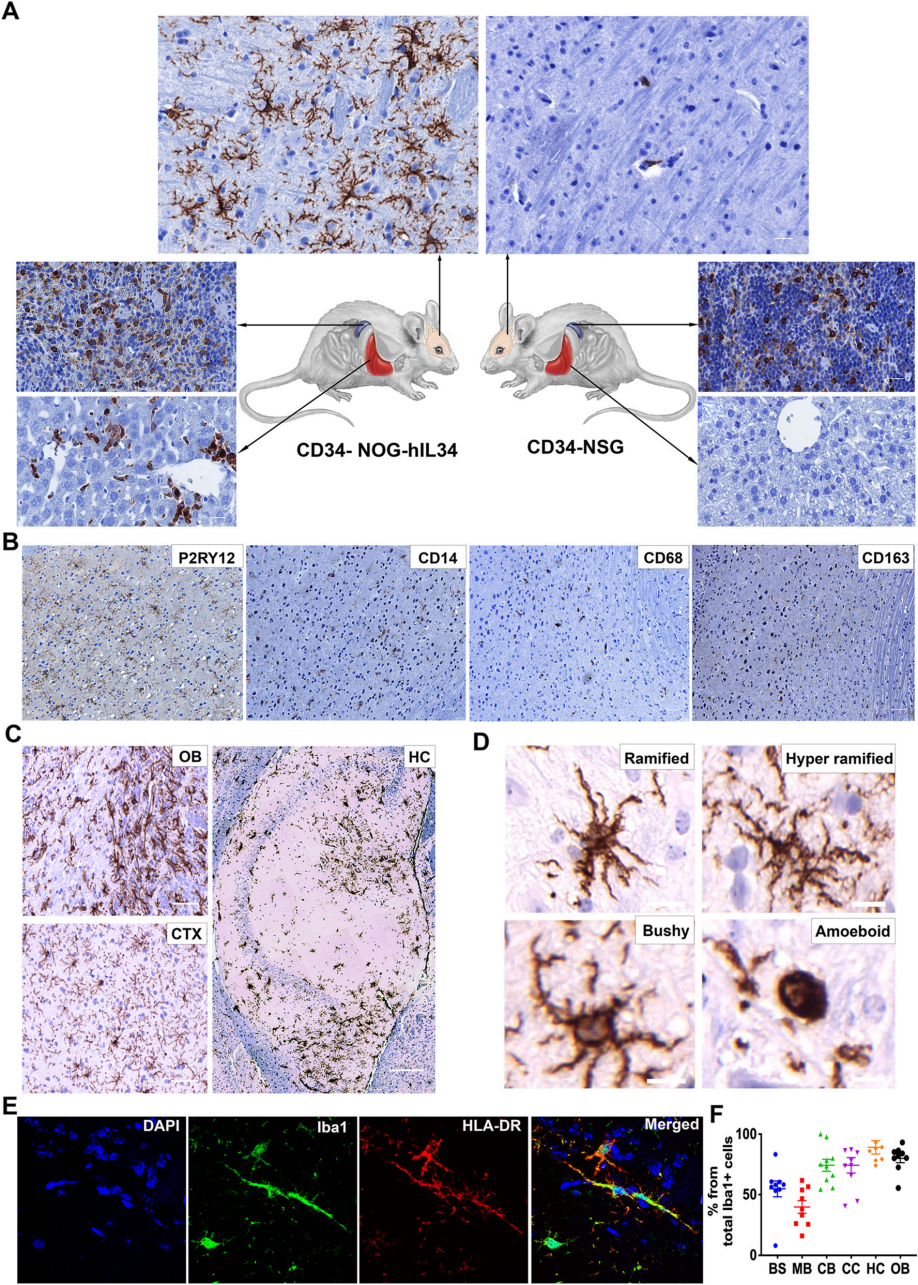
Human microglial-like cells in CD34-NOG-hIL-34 mouse brains. **a** A comparison of tissue macrophage reconstitution between CD34-NOG-hIL-34 and CD34-NSG mice. Brain sections were stained for HLA-DR, and liver and spleen for human CD68 (Scale bar 10 μm). HLA-DR+ human cells were widely distributed in brain parenchyma of CD34-NOG-hIL-34 mice. **b** Magnified views of olfactory bulb (OB, 20×), cortex (CTX, 20×) and hippocampus (HC, 10×) from CD34-NOG-hIL-34 mouse brain sections stained for HLA-DR. **c** Microglial morphology of the human cells shown at higher magnification (Scale bar 2 μm). **d** Confocal images of brain stained for HLA-DR and Iba1. **e** Quantification of HLA-DR/Iba1 double positive human microglial cells from total Iba1 positive cells (sample size described in materials and methods, multiple reagions shown on Additional file 1: Figure S5)

We next examined the brains of CD34-NOG-hIL-34 mice for the presence of human cells. Surprisingly, significant numbers of human microglial-like cells were present in the brains of CD34-NOG-hIL-34 mice (Fig. 2a,b) (Additional file 1: Figure S4), compared to CD34-NSG mice, where they were rare or absent. In CD34-NSG mice, human cells are mostly present in meninges, few in perivascular areas and rarely in parenchyma [8, 9, 33]. Tissue human macrophage engraftment was also evident in CD34-NOG-hIL-34 mice, especially with liver Kupffer cells lining sinusoid walls (Fig. 2a). Human microglial-like cells were widely distributed throughout the mouse brain regions in CD34-NOG-hIL-34 mice, with highest presence in olfactory bulb (OB), cortex (CTX), hippocampus (HC), thalamus (TH), substantia nigra (SN) and cerebellum (CB) (Fig. 2b and Additional file 1: Figure S4). Microglia mostly with ramified and some with immature compact amoeboid morphology were easily demonstrated (Fig. 2c). The presence of human microglial cells was evident from 4 months of age (data shown at 6 months). Percent human microglia from total Iba1+ cell population was determined by counting HLA-DR^+^/Iba1^+^ double positive human microglia and HLA-DR^−^/Iba1^+^ mouse microglia. Human microglial reconstitution was up to 80% of the total microglial population in select brain regions, suggesting that human microglial cells replaced their mouse counterparts (Fig. 2d,e) (Additional file 1: Figure S5). Human microglial interaction with mouse CNS revealed normal astrocyte behavior and neuronal integrity without the signs of phagocytic phenotype of human microglial cells against mouse cells, as determined by lack of colocalization of astrocytic or neuronal staining with HLA-DR+ human microglia (Additional file 1: Figure S6).

### Characterization of human microglial-like cells

**Fig. 3:**
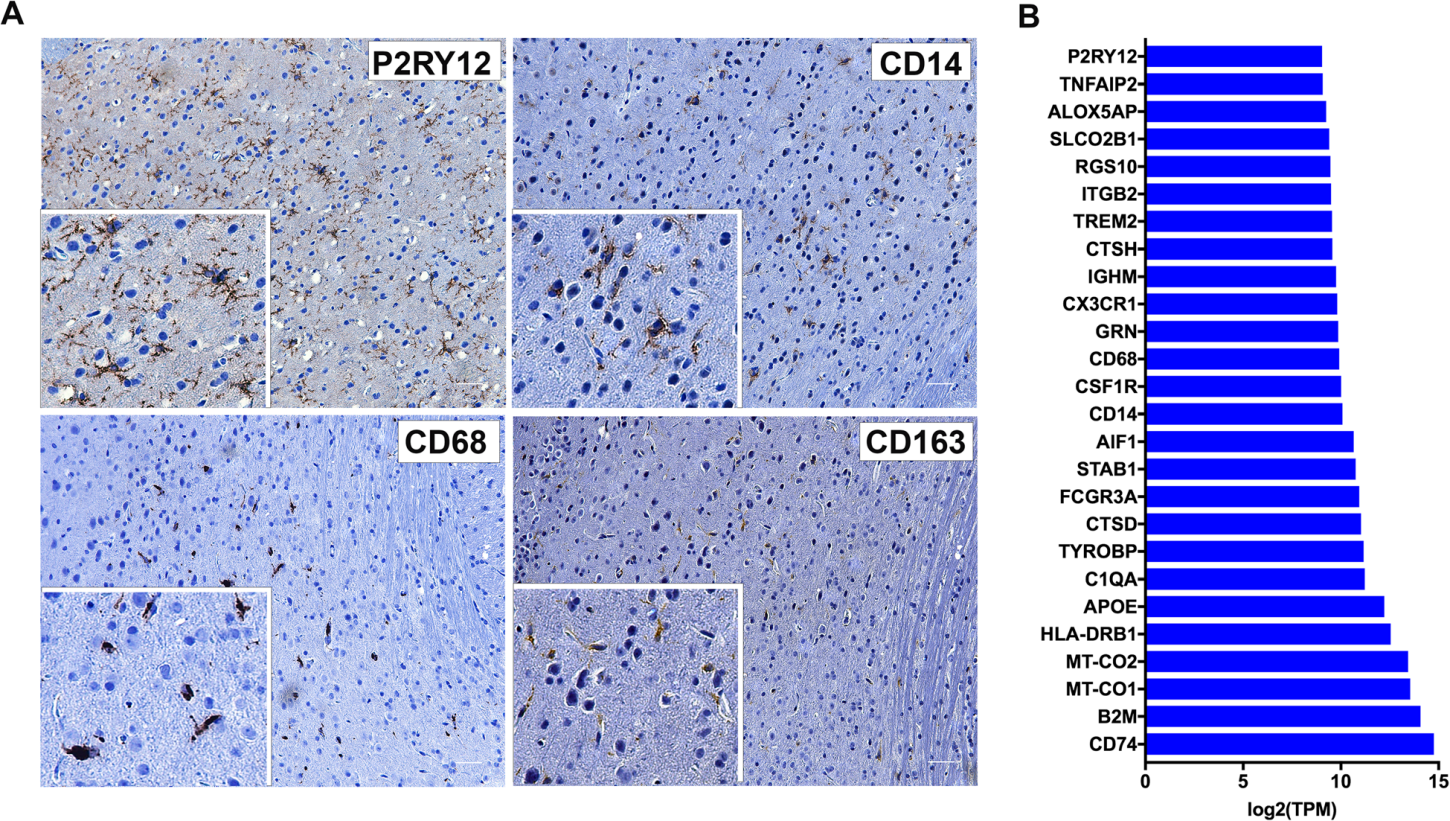
Expression of microglial markers by the human cells in mouse brain. **a** Immunohistology of 5 μm paraffin embedded CD34-NOG-IL-34 mouse brain sections stained for human microglial markers P2RY12, CD14, CD68 and CD163. Brown cells are positive for the respective protein (Magnification, 200×, Scale bar 20 μm) Inset shows 1000× magnification. **b** Transcriptomic analysis of RNA extracted from CD34-NOG-IL-34 mice brains by aligning the reads to human genome (h19) showed human myeloid specific gene expression. The graph shows gene names and expression levels of top classical microglial markers in CD34-NOG-hIL-34 mouse brains

The human HLA-DR positive cells in mouse brain not only had microglial morphology, but also positive for myeloid markers CD14, CD163, and CD68. Most importantly, the microglial-like cells populating mouse brain were positive for the putative microglial marker, purinergic receptor P2RY12 (Fig. 3a) [34]. Further, deep sequencing of brain RNA from CD34-NOG-hIL-34 mice and refined search for human genes indicated that a total of 82 human myeloid/monocyte/macrophages/microglia related genes were expressed by the human cells in CD34-NOG-hIL-34 mice. The highest expression was noted for MHC Class II (CD74) and Class I (B2M). Expression of the classical macrophage/microglial markers [35], such as AIF1 (IBA1), CD14, CD68, CSF1R, ITGAM (CD11b), P2RY12, CX3CR1, Trem2, TMEM119 were also noted. A spectrum of cytokines and chemokines secreted by microglia were detected, such as chemokine ligand (CCL)-2, tumor necrosis factor (TNF), IL-6, C-X-C motif chemokine ligand (CXCL) 8, IL-8, IL-10, IL-1A, CXCL10 (interferon gamma induced protein 10). The transcription factors PU.1 (SPI 1), ETV5, and apolipoprotein E (APOE) critical for microglial health and function were also found (Fig. 3b) (Additional file 1: Table S2).

### HIV-1 infection of the human microglial mouse

**Fig. 4:**
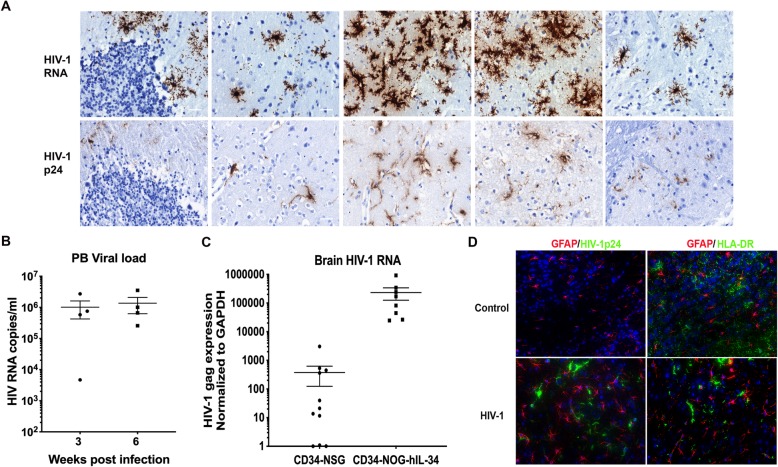
HIV infection in the humanized mouse brain. **a** RNAScope® assay for detecting HIV-1 RNA, using antisense probe V-HIV1- Clade-B, revealed single brown dots or cluster of dots. Immunohistology of brain regions showing the presence of HIV-1p24+ infected cells in corresponding regions. Original magnification of 400×, Scale bar 10 μm. **b** Peripheral viral load was determined by a COBAS Amplicor System after three and 6 weeks post infection. Each symbol represents an individual infected mouse. **c** Comparison of brain viral RNA levels determined by semi-nested RT-PCR from CD34-NSG and CD34-NOG-hIL-34 mice. **d** Immunofluorescence staining demonstrating mouse astroglia (GFAP, red) in control and HIV-1 infected, with astrogliosis near in HIV-1p24 positive (green) human microglia. Adjacent sections stained for human microglial cells (HLA-DR+ green) and astrogliosis (GFAP, red) indicating the presence of human microglial cells in the same brain region. Original magnification of 400×

Next, when mice, at 6 months age, were infected with an R5-tropic HIV-1_ADA_ strain injected intraperitoneally, HIV infection was consequently established with viral loads in peripheral blood at ~ 10^6^ RNA copies/ml along with infection in secondary lymphoid organs (Fig. 4b) (Additional file 1: Figure S7). A slight depletion of CD4+ T cells was observed by 6 weeks of infection as seen in our previous reports [7–9, 28, 36]. Peripheral infection resulted in a robust infection of human microglia which was easily detected (Fig. 4a) (Additional file 1: Figure S8). Infected cells were found throughout the mouse brain regions, with highest levels of infection in OB, HC, TH and CTX. RNAScope® technique allowed to see clear infected human cells and extracellular viral particles released from infected cells. Brains of CD34-NOG-hIL-34 mice had 3–4 log_10_ times (10^6^ vs 10^2^) higher HIV viral load compared to the CD34-NSG model reconstituted with only human immune system having similar peripheral viral loads (Fig. 4c) (Additional file 1: Table S1). Reactive astrocytes were readily detected, at or near, HIV-1 infected microglia (Fig. 4d).

### The HIV-1 infected microglial transcriptome

**Fig. 5:**
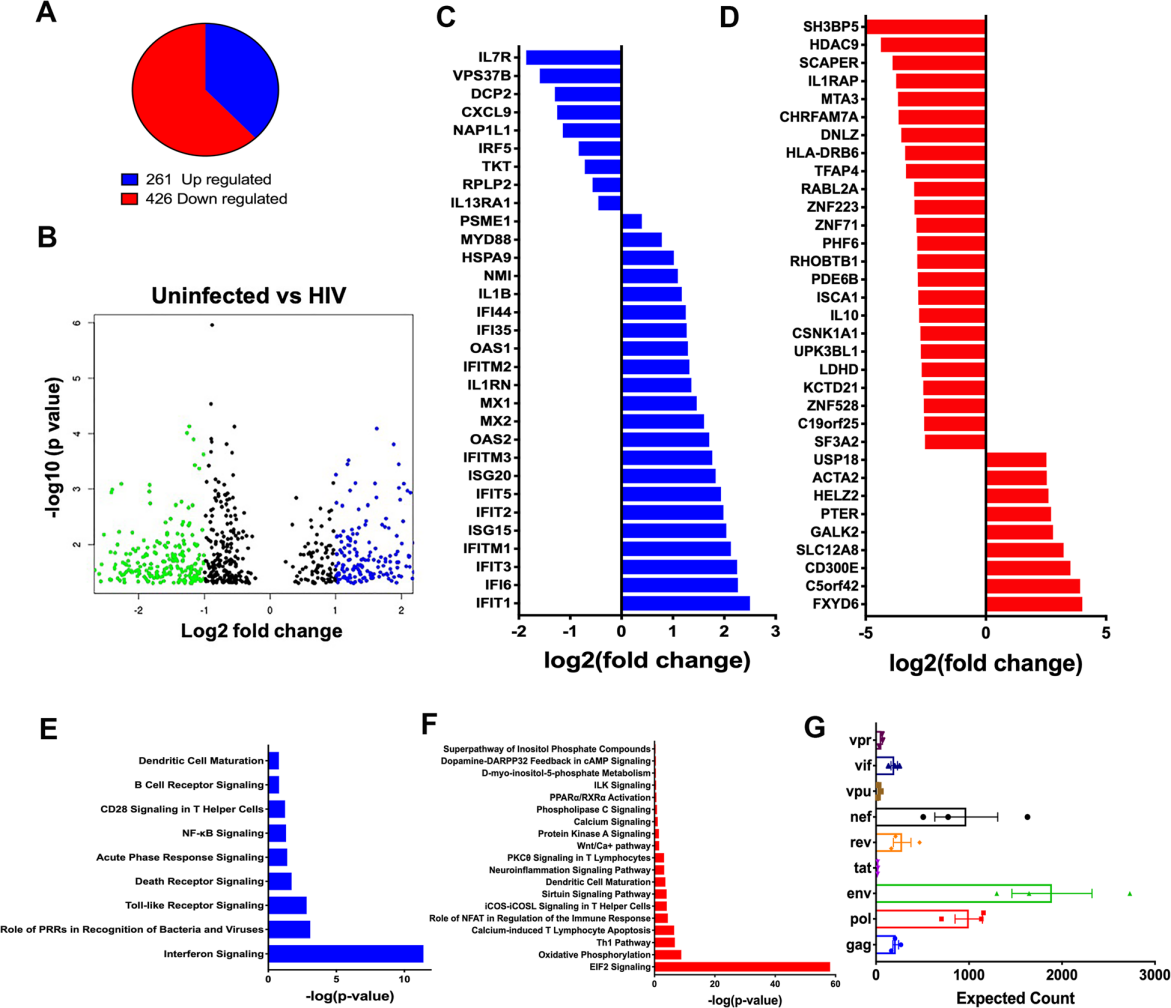
Transcriptional changes in the brain tissues of CD34-NOG-hIL-34 with HIV-1 infection. **a, b** Alignment of reads to human genome (h19) comparing uninfected and HIV-1 infected CD34-NOG-IL-34 mice found 687 differentially expressed genes (DEG), and the pie chart (**a**) and the volcano plot (**b**) shows the proportion and fold change of upregulated and down-regulated genes, respectively. **c, d** Log fold change of top ranking differentially expressed human genes for uninfected vs HIV infected CD34-NOG-hIL-34 mice in human microglia and in brain. **e, f** Cellular pathways involving upregulated human genes (261) were related to interferon signaling, PRR and TLR signaling (**e**), and downregulated human genes (426) were highly associated with pathways of EIF2 signaling and oxidative phosphorylation (**f**). **g** Unmapped reads mapped to HIV-1_ADA_ genome indicated the expression levels of different HIV-1 genes in infected mouse brain

Comparison of human specific genes in HIV infected CD34-NOG-hIL-34 mice to uninfected showed a significant downregulation of majority of the transcripts (62%, 426 genes) among the total differentially expressed genes (687genes) (Fig. 5a, b). The top ranking upregulated and down regulated genes were selected to plot Fig. 5c and d, respectively. The entire list of genes that were differentially expressed are given in supplementary table for further understanding of the same (Additional file 1: Table S3 and Table S4). Majority of the downregulated genes took part in eukaryotic translation initiation factor 2 (EIF2) signaling whereas upregulated genes were part of interferon signaling (Fig. 5c-f). Most significant human genes upregulated by HIV infection were anti-viral defense genes related to interferon (IFN) pathways such as IFIT1–5, ISG15, MX2, OAS1, OAS2, ISG20 [23, 24]. The other pathways and related genes that were upregulated include pattern recognition receptors and toll-like receptor (TLR) that recognize viruses (TLR8, IFIH1, MYD88, OAS1,2&3). The increased expression of TLR8 and myeloid differentiation factor 88 (MYD88) are linked to the activation of NF-κB transcription factor induced genes (BCL10, IL1RN, IL1B, MALT1) and MAPK (MAPK12) [37]. Mapping the reads from infected CD34-NOG-hIL-34 mice against HIV-1 genome showed significant counts of several HIV-1 genes with gag, nef and env (Fig. 5g), that were not detected previously in CD34-NSG mice brains with human astrocytes and limited infection in brain [10].

## Discussion

We report, for the first time, a human microglial mouse developed from human bone-marrow mouse chimeras, engrafted as newborns intrahepatically with umbilical cord blood derived CD34+ HSPC. Previously, several attempts were reported in reconstituting mouse brain post-natally with HSPC-derived microglia [38–41]. The microglial reconstitution of mouse brain was mostly achieved by the creating a niche by depletion of intrinsic mouse microglia [38], however, these peripherally derived microglial-like cells were distinct in identity from microglia [41]. In HSPC-transplanted humanized mice reconstituted with human hematolymphoid system, there is limited infiltration of human myeloid cells into the mouse brain, mostly in meninges [8, 9, 33, 42]. These cells expressed the microglial marker P2RY12, but did not have microglial morphology [43]. Even with high peripheral reconstitution of human myeloid in human CSF-1 transgenic immunodeficient mice, human microglial cells were not reported in mouse brain [19]. Our findings clearly support the fact that hIL-34 was the key player in the human microglial development in humanized mice. In HSPC transplanted hIL-34 NOG mice, bone marrow-derived monocytes migrated into the mouse brain and differentiated into microglia in presence of human IL-34. Alternatively, human HSPC could also seed the mouse brain very early after transplantation due to conditioning by irradiation and continue to generate human microglial cells in situ [44, 45].

Long-term efforts to reconstitute human microglia in mice have previously attempted with limited success [46]. There are many caveats with the development of microglia form HSPC because of their origin and ontogeny. Microglia are the resident macrophages of the CNS providing developmental support and immune brain protection. The cell’s precise origin, ontogeny and identifying markers in human are still remain elusive [47–49]. In adult brain, either in normal or disease state, it is unclear whether the microglia originate solely from cells present in the brain from fetal origins or if there is further input into CNS from HSPC derived myeloid cells [50–52]. While monocytes enter the CNS under steady state and diseased conditions and are transformed into microglial-like cells their “true identity” remains uncertain [41, 50, 53]. Mouse-to-mouse transplant experiments show that donor HSPC can produce microglial-like cells in mice depleted of resident microglia by radiation, chemotherapy, or through cell-specific suicide genes [38, 54]. Microglial ablation scheme using PLX5622, a selective inhibitor of CSF1R, demonstrated that all repopulated microglia were derived from the proliferation of the few surviving microglia [55]. To date, human microglial reconstitution in immune deficient mice was not efficient, regardless of the approach used [46].

IL-34 is not a sole factor responsible for this microglial reconstitution. The regulation of peripheral macrophage ingress into the fetal brain and microglial-like cell differentiation and expansion is facilitated by a gradient of signaling mediators. These include, but are not limited to, matrix metalloproteinases, semaphorins/netrins, monocyte chemoattractant protein-1 (MCP1), macrophage inflammatory protein1 aplha (MIP-1α), ligands of CSF-1R, the fractalkine (CX3CL1)/receptor1 pathway, receptor tyrosine kinase/vascular endothelial growth factor receptor 1 (VEGFR1) interactions, and others [56]. We cannot exclude hIL-34 interactions with the receptor-type protein-tyrosine phosphatase zeta (RTPTP-ζ), a cell surface chondroitin sulfate proteoglycan identified as a second receptor for IL-34, important for cells’ ramification [57]. The detailed underlying mechanisms for the successful microglial-like cell reconstitution of mouse brain beyond human IL-34 requires further investigation. Such studies would allow an understanding of what mouse factors influence such brain cell ingress and reconstitution. Adding to such complexities is the fact that human microglial reconstitution in the mouse brain has regional variation. Although human IL34 is constitutively expressed in all cells in the hIL-34 transgenic mice such heterogenous distribution showed high cell density in the hippocampus, olfactory tubercle, basal ganglia, cortex, thalamus and substantia nigra [58]. Regional variation in microglial distribution is also evident in rat and human brains [59, 60]. In this model human microglia appear to replace mouse microglia. How such “putative” replacement plays a role in brain function is yet to be determined.

Humanized mouse models reconstituted with human hematolymphoid cells have been used to study highly relevant aspects of HIV replication, pathogenesis, therapy, transmission, prevention, and eradication [Reviewed in [4, 61, 62]]. These models were also evaluated for the studies of NeuroHIV and brain viral reservoirs [11, 63, 64]. However, lack of human microglia and astrocytes in the mouse brain limits humanized mouse models to study HIV induced brain pathology and to test HIV eradication strategies targeting brain viral reservoirs. Unique humanized mouse models reconstituted with human blood and brain glial cells were developed within our laboratories [10] to mirror human specific antiviral responses that affect HIV brain infections. Human microglia are a major HIV-1 CNS reservoir. We and others explored divergent means to reconstitute the mouse brain with human microglia for studies of HIV-1 infection [8, 42, 43, 65, 66]. Now using hIL-34 transgenic mice, we successfully reconstituted mouse brain with human microglia and established persistent viral infection. Robust infection of microglial cells was observed independent of peripheral infection. It was suggested that peripheral blood monocytes differentiated in presence of IL-34 had increased resistance to HIV-1 infection over macrophage colony stimulating factor (MCSF) derived macrophages [67]. In contrary, both MCSF and IL-34 enhance HIV-1 infection in human microglia in vitro [68]. In SIV infected rhesus macaques IL-34 expression was not increased, unlike MCSF, in macrophages accumulating perivascularly and within the nodular lesions in brains with or without encephalitis. Both MCSF and IL-34 were shown to upregulate CD163 in primary monocytes, a marker for type 2 activation of macrophages [69]. The human microglia in hIL-34 transgenic mouse brain expressed CD163 as detected by both immunohistology and RNAseq analysis, nevertheless, we cannot compare the CD163 expression levels to microglia developed in the absence of IL-34 as there are no microglia in the brains of CD34-NSG mice. The infection in mouse brain was higher in OB, HC, TH and CTX and levels of infection correlated with the higher density of human microglial cells in these regions. In human brain infection is predominantly seen in globus pallidus, substantia nigra and dentate nucleus [70]. Brain imaging studies revealed signs of inflammation and dysmorphology mainly in cortical and subcortical regions, white matter, and basal ganglia deep grey matter. Impairments in memory and hippocampal function, motor deficits with reduced basal ganglia and thalamic volumes were reported [71–74]. HIV infection was also associated with reduced olfactory function with significant decrease in odor identification scores across time [75–77]. Differential gene expression analysis was used to determine the functionality of brain engrafted human microglia in response to HIV infection. The major human cell type in the mouse brain was human microglia, hence, the human genes detected were associated to these cells. There is increased infiltration of human macrophages into the mouse brain mainly in meninges and few in perivascular areas [8], however they are less in number compared to the human microglial cells in the brain parenchyma with infection. We could segregate species-specific responses in our complex human-mouse chimeras [10]. There is a significant increase in the expression of anti-viral defense genes related to interferon signaling (IFI and IFIT families). Toll-like receptor signaling and NF-κB signaling were also upregulated indicating activation of innate immune response, increased inflammation and immune cell regulation, as host defense mechanisms [78–81]. Transcriptomic profiles of the brains of HIV infected patients and SIV infection macaques also displayed significant upregulation of interferon signaling and inflammation related pathways [24, 82–85]. Among down regulated pathways, genes related to eIF2 signaling were affected with HIV infection. Evidence suggests that host cell protein synthesis is modulated during HIV infection and the effect on host protein synthesis can be garnered from the inhibition of PKR induced eIF2 phosphorylation by HIV tat so that the synthesis of the viral proteins occur freely [86, 87]. eIF2 was found affected in patients suffering from neurodegenerative diseases such as Alzheimer’s, Parkinson’s and Huntington’s disease [88]. Further analysis is required to understand the effect of human microglial reconstitution on mouse brain cells and the infection induced neuropathogenesis. In this current study we presented the phenotype of human microglial cells and their response to acute systemic HIV infection. The presence of human microglia and HIV-1 infection showed mild changes in mouse transcriptomics. Further refined analysis of mouse genes is needed to understand the effects of human microglia on neuronal functional and structural changes and deficiencies induced by HIV-1 infection of human cells. Brain pathogenesis assessment require chronic infection, extended analyses and multi-modalities approach as with our previous report using behavior and imaging evaluations [7].

## Conclusion

All together we created a humanized mouse that contained both a human immune system and human brain glial cells. The strength of this model allows it to be used to mimic innate immune activities of the CNS and its interface with peripheral adaptive immunity. The model also offers a template for the studies of HIV-1 infection, neuropathogenesis, therapeutics and potential reservoir sites within the CNS. In this manner the model offers new unchartered opportunities to study disease states of the human brain that were not possible in any past time. Such new opportunities will surely open up pathways towards therapeutics and disease cure strategies.

## Additional file

Additional file 1: **Figure S1.** Integrated site of the transgene in NOG mouse genome. **Figure S2.** Human IL-34 expression in different regions of mouse brain. **Figure S3.** Comparison of human immune cell reconstitution in NSG and NOG-hIL-34 mice. **Figure S4.** Human microglial reconstitution in mouse brain. **Figure S5.** Human and mouse glial cell distribution in mouse brain. **Figure S6.** Mouse neuronal cell interaction with human microglial cells. **Figure S7.** Establishment of systemic HIV infection in CD34-NOG-hIL-34 mice. **Figure S8.** HIV-1 infection in mouse brain. **Figure S9.** Flowchart of deep sequencing analysis. **Table S1.** Plasma viral loads of HIV-1 infected humanized NSG mice. **Table S2.** List of genes expressed by microglia. **Table S3.** HIV induced transcriptional changes evaluated at 8 months of age (hg19)- Upregulation. **Table S4.** HIV induced transcriptional changes evaluated at 8 months of age (hg19)- Downregulation. (PDF 1822 kb)

## Abbreviations

AF: Alexa Fluor
APC: Allophycocyanin
APOE: Apolipoprotein E
BH: Benjamini-Hochberg
BV: Brilliant Violet
cART: Combination anti-retroviral therapy
CB: Cerebellum
CCL: Chemokine ligand
CD: Custer differentiation
CD34-NOG- hIL-34: CD34 cell transplanted NOG-IL-34
CIEA: Central Institute of Experimental Animals
CNS: Central nervous system
CSF-1: Colony stimulating factor-1
CSF1R: CSF1receptor
CTX: Cortex
CX3CL1, VEGFR1, RTPTP-ζ: Receptor-type protein-tyrosine phosphatase zeta
CX3CR1: C-X3-C Motif Chemokine Receptor 1
CXCL: C-X-C motif chemokine ligand
DAB: 3,3'-Diaminobenzidine
EDTA: Ethylene diamine tetraacetic acid
EIF2: Eukaryotic translation initiation factor 2
FACS: Fluorescence activated cell sorting
FITC: Fluorescein isothiocyanate
HC: Hippocampus
hIL-34: Human IL-34
HIV-1: Human Immunodeficiency Virus-1
HRP: Horse radish peroxidase
HSPC: Hematopoietic stem progenitor cells
IFN: Interferon
IL-34: Interleukin-34
ITGB2: Integrin Beta 2
MCP-1: Monocyte chemoattractant protein-1
MCSF: Macrophage colony stimulating factor
MIP-1α: Macrophage inflammatory protein1 aplha
Ms: Mouse
NOG-hIL-34: NOD.Cg-*Prkdc*^*scid*^*Il2rg*^*tm1Sug*^Tg(CMV-IL34)1/Jic
NSG/NOG: NOD/Scid IL2Rγ−/−
OB: Olfactory bulb
P2RY12: Purinergic Receptor P2Y
PCR: Polymerase chain reaction
PE: Phycoerythrin
Rb: Rabbit
SEM: Standard error of the mean
SN: Substantia Niagra
TCID_50_: Tissue Culture Infectious Dose 50
TG: Transgene
TH: Thalamus
TLA: Targetted locus amplification
TLR: Toll like receptor
TNF: Tumor necrosos factor
TPM: Transcripts Per Kilobase Million
Trem2: triggering Receptor Expressed On Myeloid Cells 2
UNMC: University of Nebraska Medical Center
