# Supplementary material for: Human Interleukin-34 facilitates microglia-like cell differentiation and persistent HIV-1 infection in humanized mice
Source: Mol Neurodegener. 2019 Mar 5;14:12. doi: 10.1186/s13024-019-0311-y (PMC6399898; doi:10.1186/s13024-019-0311-y)
Supplement: Supplementary file 1 — Figure S1. Integrated site of the transgene in NOG mouse genome. Figure S2. Human IL-34 expression in different regions of mouse brain. Figure S3. Comparison of human immune cell reconstitution in NSG and NOG-hIL-34 mice. Figure S4. Human microglial reconstitution in mouse brain. Figure S5. Human and mouse glial cell distribution in mouse brain. Figure S6. Mouse neuronal cell interaction with human microglial cells. Figure S7. Establishment of systemic HIV infection in CD34-NOG-hIL-34 mice. Figure S8. HIV-1 infection in mouse brain. Figure S9. Flowchart of deep sequencing analysis. Table S1. Plasma viral loads of HIV-1 infected humanized NSG mice. Table S2. List of genes expressed by microglia. Table S3. HIV induced transcriptional changes evaluated at 8 months of age (hg19)- Upregulation. Table S4. HIV induced transcriptional changes evaluated at 8 months of age (hg19)- Downregulation. (PDF 1822 kb) [file 13024_2019_311_MOESM1_ESM.pdf]

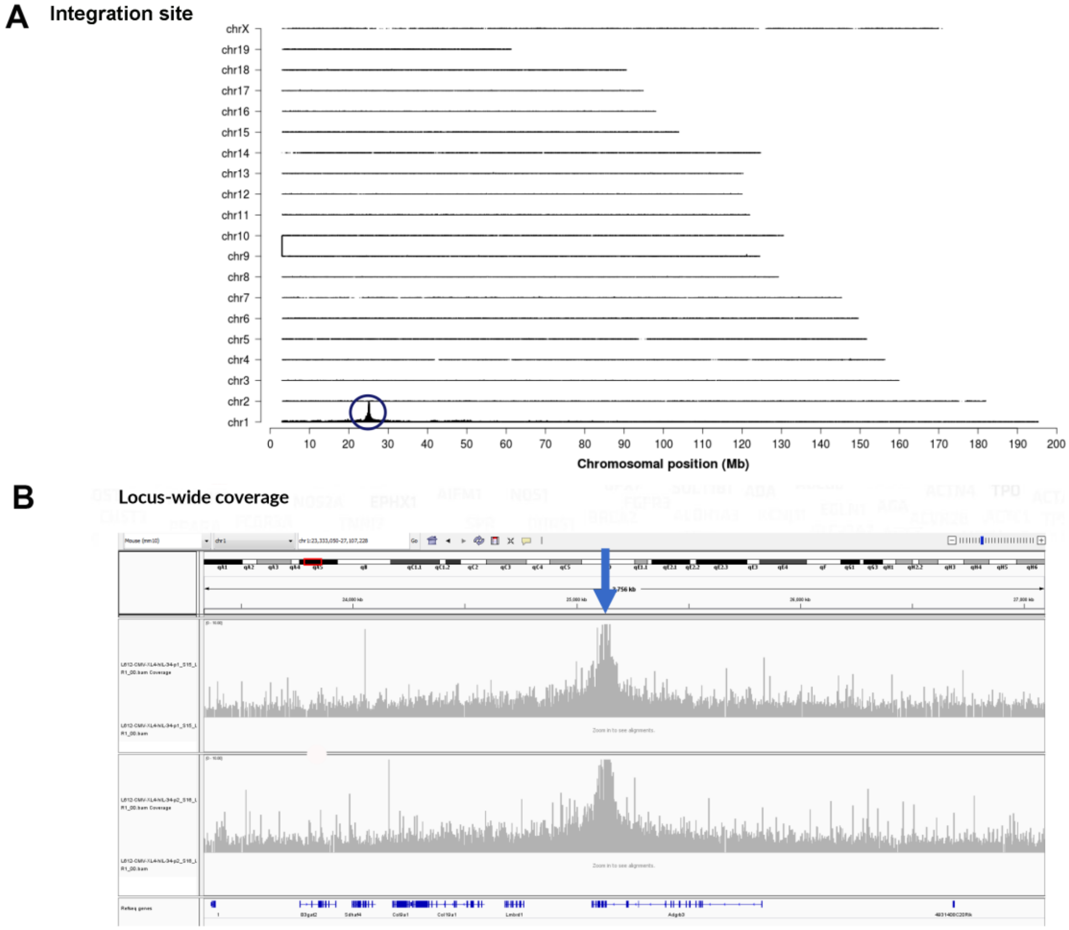

C

5' integration site:
chr1:25,127,822 (tail) fused to TG:6,459 (tail) with one inserted base
TTTTATAAACAGACAGACAGAAACACTAGTATACTCAGTTACCAATATATGTGCATTTATTTAAAGATCAACTCAAG
ACTTGGTATGCTTTTGACACTCACCAGTCACAGAAAAGCATCTTACGGATGGCATGACAGTAAGAGAATTATGC

3' integration site:
TG:6,459 (tail) fused to chr1:25,127,823 (head) with one inserted base
CATGCCATCCGTAAGATGCTTTTCTGTGACTGGTGAGTCTTTATAAACTCGAAAGTAGAAACCATAGTATCTGTAAT
GCCAATAAGGTGTTTTACAAAAACTTG

Figure S1: Integrated site of the transgene in NOG mouse genome. (a) The transgene has integrated in mouse chromosome 1 (chr1) and the chromosome position is indicated on the x axis. No structural rearrangements were found in the host genome around the integration site. (b) TLA sequence coverage across the transgene integration locus; mouse chr1:23,333,050-27,107,228. Sequence coverage (in grey) generated with primer set 1 (top) and primer set 2 (bottom). The blue arrow points toward the breakpoint sequences identified with both primer sets. Y-axis is limited to 10x. **(c)** Breakpoint sequences/reads identified marking the transgene integration. The coverage profile shows that no genomic rearrangements have occurred in the region of the integration site. Transgene has integrated in mouse chr1:25,127,822-25,127,823. According to the refseq integration of gene is in the intronic region of *Adgrb3*.

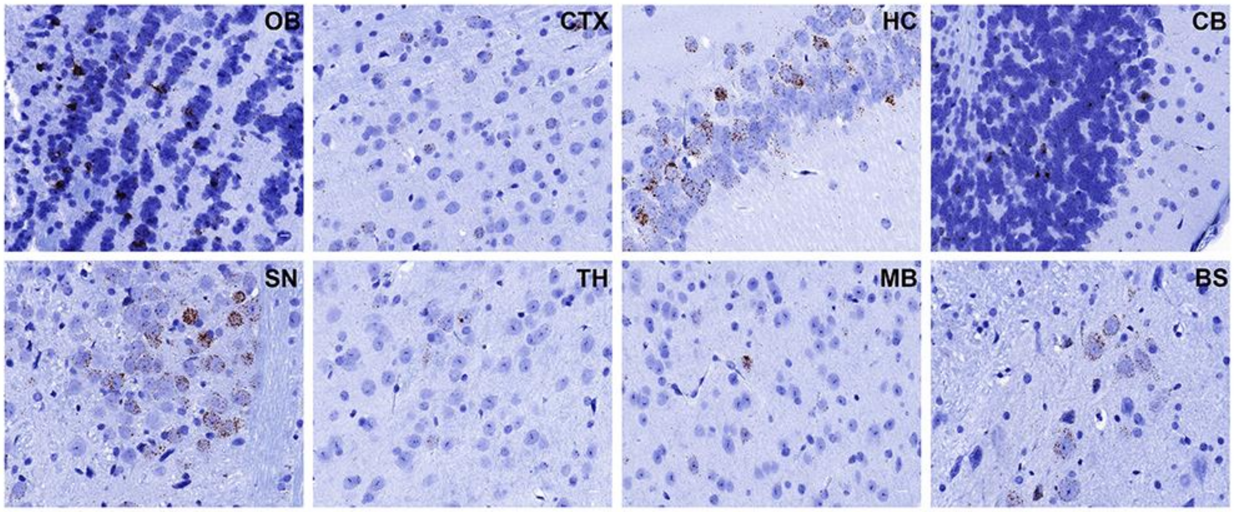

Figure S2: Human IL-34 expression in different regions of mouse brain. RNAScope® assay for detecting human IL-34 RNA revealed single brown dots or cluster of dots in 5 μm thick brain sections using antisense probe Hs-IL34-NoXMm targeting 38-1774 base pair of human IL-34. NOG mice showed no signals corresponding to the presence of human IL-34. Images are 40x magnification (scale bar- 10 μm). Analysis was carried out in a minimum of 4 samples and representative images from one mouse brain are displayed.

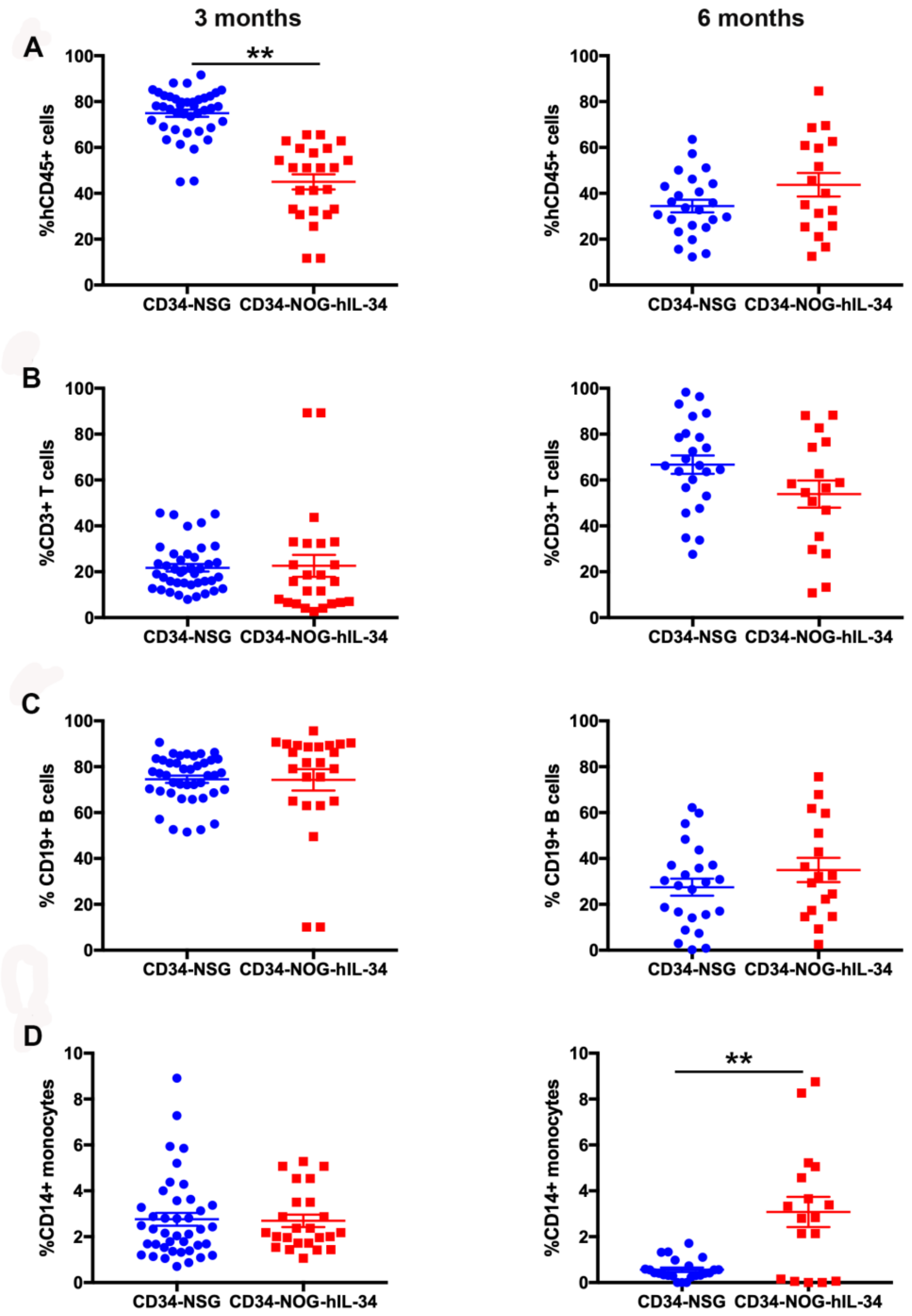

Figure S3: Comparison of human immune cell reconstitution in NSG and NSG-hIL-34 mice. Mice were transplanted at birth with HSPC. Flow cytometry analysis of peripheral blood at 3 months (right) and 6 months (left) post transplantation. **a,b,c,d**, are plots showing the percentages of human CD45, CD3, CD19 and CD14 positive cells, respectively. CD3, CD19 and CD14 positive cells from human $CD45^+$ gate. Each symbol represents an individual mouse. **p<0.001

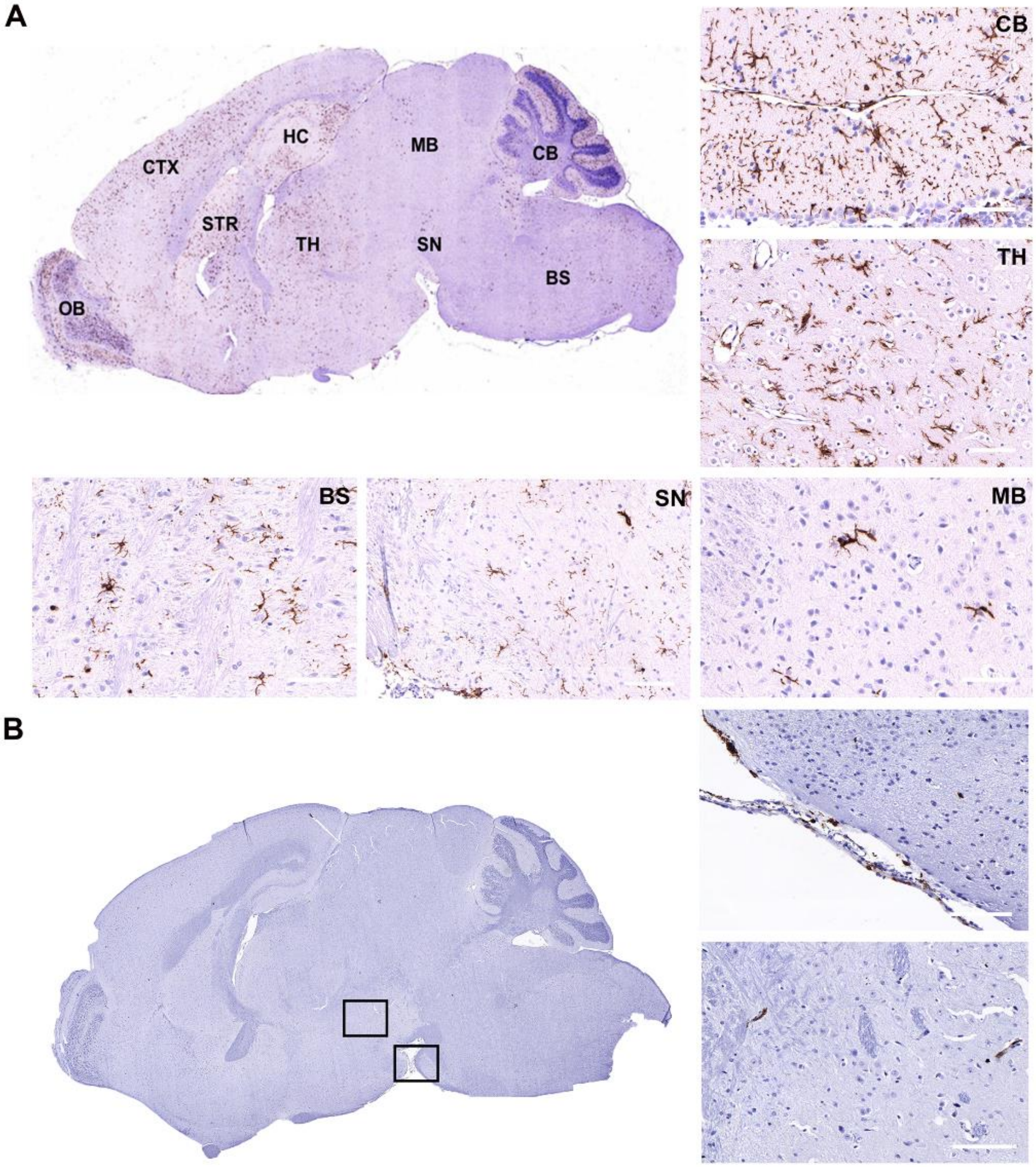

Figure S4: Human microglial reconstitution in mouse brain. 5 µm thick paraffin embedded brain sections were stained for human MHC class II (HLA-DR). **(a)** Representative CD34-NOG-hIL-34 mouse brain section is shown at 2x magnification to give an overall view of human microglial distribution throughout the brain regions: olfactory bulb (OB), cerebral cortex (CTX), hippocampus, midbrain (MB), cerebellum (CB), striatum (STR), hippocampus (HC), substantia niagra (SN), thalamus (TH) and brain stem (BS) regions, captured by a VENTANA iScan HT at an original magnification

of 200x. Magnified views (20x objective) of brain regions (OB, CTX and HC are shown in Figure 2B) showing HLADR+ cells with microglial morphology. **(b)** Representative CD34- NSG mouse brain section (2x) demonstrating lack of human microglial cells. HLA DR+ cells in perivascular (top) and meningeal (bottom) areas (boxed) are shown at magnified views (20x), Scale bar-20 µm.

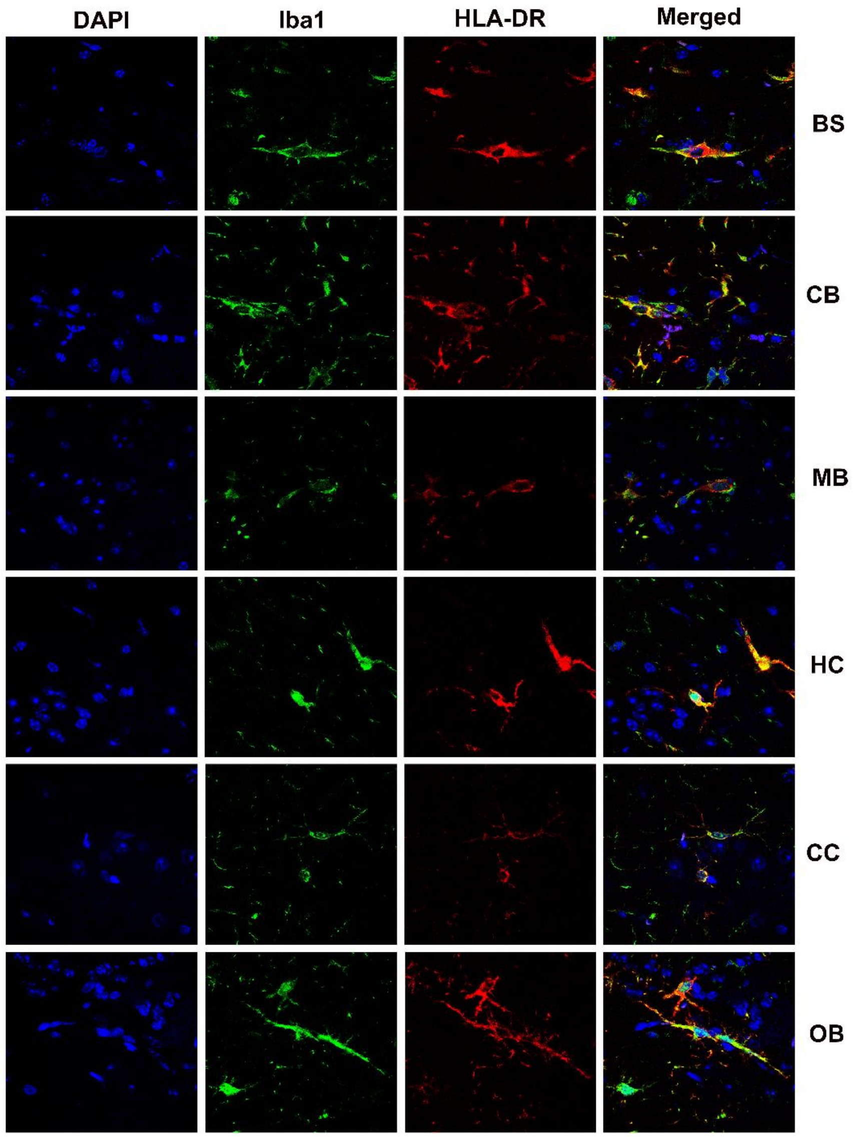

Figure S5: Human and mouse glial cell distribution in mouse brain. Sagittal section of paraffin-embedded brain tissue stained for human MHC class II (HLA-DR, red) and microglial cells (Iba1, green). Cells are identified as HLA-DR/Iba1 double positive human microglia and Iba1 single positive mouse microglia. Original confocal images were collected by a Zeiss 710 system at 630x magnification. Images from one brain sample are shown. For quantification of the number of human microglial cells, a minimum of 2-4 selected field of views of the same brain region under 400× magnification were counted for HLA-DR+/Iba1+(human microglia) and Iba1+ (mouse microglia) using Nuance Multispectral Tissue Imaging system (CRi, Wobum, MA), and shown as percentage human microglia from total Iba1+ cells in Figure 2F.

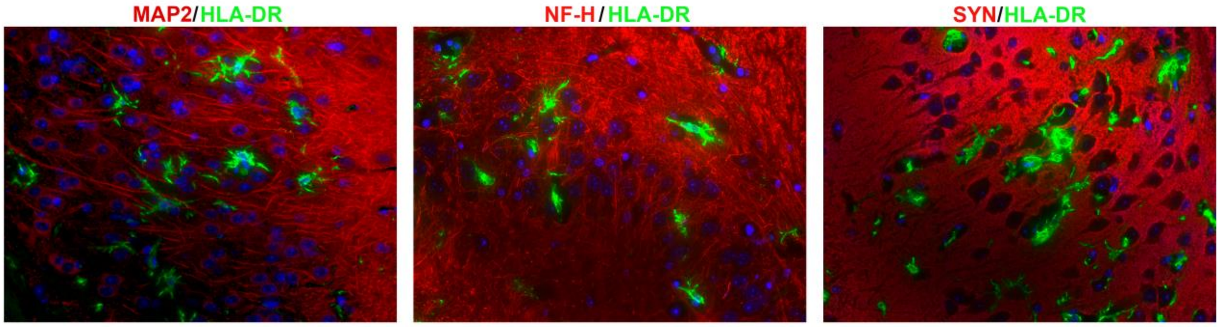

Figure S6: Mouse neuronal cell interaction with human microglial cells. Sagittal section of paraffin-embedded brain tissue stained for human MHC class II (HLA-DR, green) and mouse neuronal markers MAP-2, neurofilament H (NF-H) or synaptophysin (SYN) (green). Human microglial cells were not indicating phagocytic phenotype in response to foreign species environs. Original magnification 400x; Representative pictures from n=4 stained for the neuronal markers.

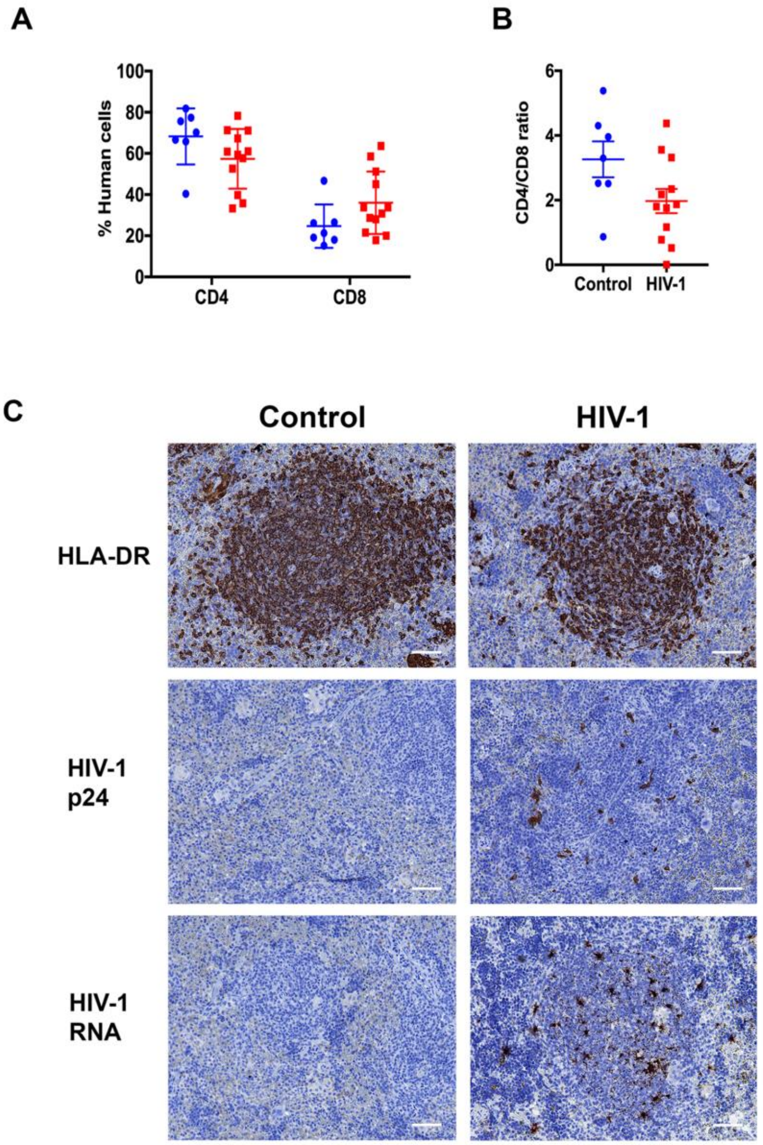

Figure S7: Establishment of systemic HIV infection in CD34-NOG-hIL-34 mice., **(a,b)** Flowcytometric analysis of splenocytes for human CD4 and CD8 positive T lymphocytes. Percent positive cells are shown in **a** and CD4/CD8 ratio in **b,** for HIV+ (n=12, red) and the control (n=7, blue) groups. At 6 weeks post infection, splenic CD4+ T lymphocyte population was not decreased significantly with HIV-1 infection. **(c)** Immunohistology of spleen sections show HLA-DR+ cells (brown) and the presence of HIV-1-infected cells (HIV-1 p24+, brown). RNAScope® assay for detecting HIV-1 RNA revealed single brown dots or cluster of dots in 5µm thick spleen sections. Uninfected mice showed no signals corresponding to the absence of viral RNA. Images were captured by a Nuance multiplex system at an original magnification of 200x, Scale bar -20 µm.

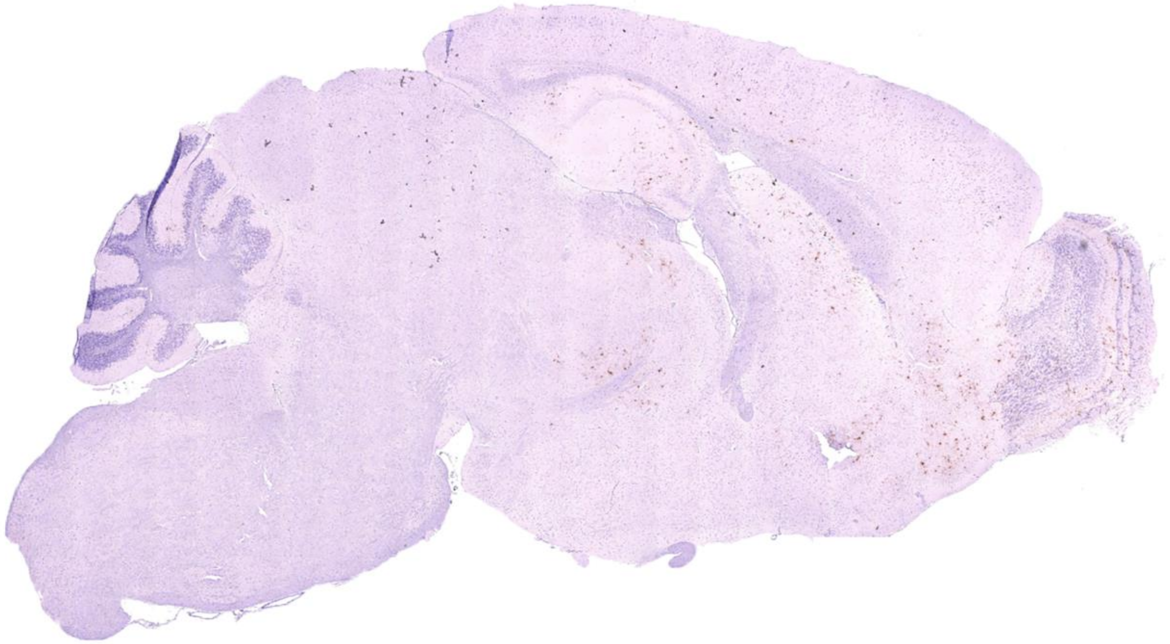

Figure S8: HIV-1 infection in mouse brain. Representative CD34-NOG-hIL-34 mouse brain section stained for HIV-1p24 (brown) is shown at 2x magnification to give an overall view of HIV-1 infection throughout the mouse brain. Image was captured by a VENTANA iScan HT at an original magnification of 400x. Infection was highest in OB, CTX, and HC.

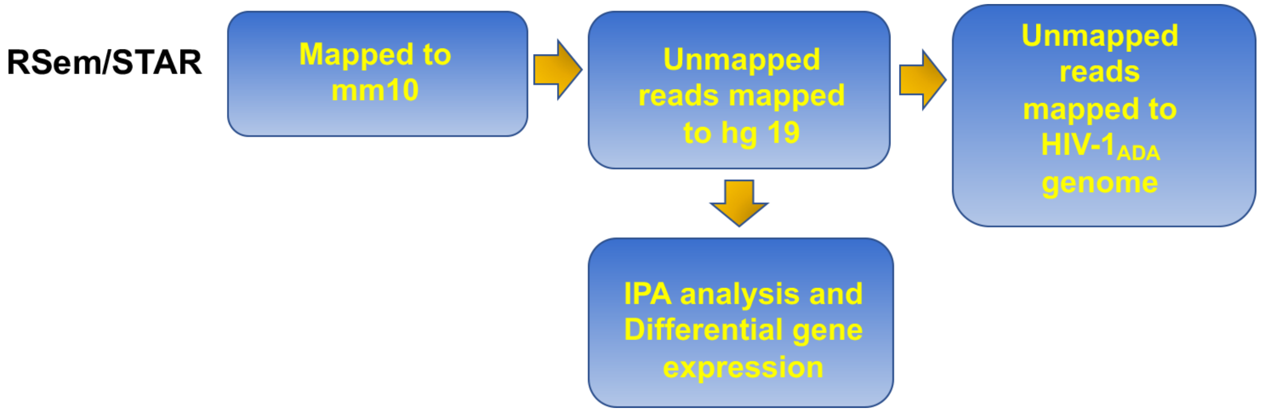

Figure S9: Flowchart of deep sequencing analysis. The reads were aligned with STAR-2.5.3a and quantified by RSEM 1.2.21 to mouse reference genome, GRCm38.p3, with default parameters, using Ensemble annotations. The unmapped reads for CD34- NOG-hIL-34 samples were further aligned to human reference genome, GrCh37 using the same pipeline and comparison analysis was performed between uninfected and HIV-1 infected samples. The reads that didn't align to human were further aligned to the HIV-1_{ADA} genome. The gene and transcript abundance were measured as Transcripts Per Kilobase Million (TPM) values. Data were filtered to include only protein coding genes. This filtered subset of genes was then used to examine the differential expression of various genes in the R statistical software environment with packages from Bioconductor and identify pathways using Ingenuity pathway analysis.

Supplemental table 1

Plasma viral loads of HIV-1 infected humanized NSG mice.

	Mouse numbers	HIV-1 RNA copies/ ml
6 weeks	3475	810600
	3476	333900
	3478	190680
	3479	310800
	3473	703500
	3485	378000
3 weeks	3463	<20
	3464	2184
	3474	1646
	3471	8841
	3469	220500
	3467	<20

Supplemental Table 2 . List of genes expressed by microglia

Gene	TPM	Log2 (TPM)
B2M	17240.7025	14.0735309
MT-CO1	11927.96	13.5420597
MT-CO2	11092.525	13.4373002
HLA-DRB1	5978.9075	12.5456662
APOE	4766.6875	12.2187713
C1QA	2387.045	11.22101
TYROBP	2290.3425	11.1613476
CTSD	2085.585	11.0262364
FCGR3A	1955.7825	10.9335302
STAB1	1723.205	10.7508786
AIF1	1613.7525	10.6562036
CD14	1081.4125	10.0787012
CSF1R	1034.5725	10.014819
CD68	963.7575	9.91252637
GRN	930.5725	9.86197474
CX3CR1	900.0075	9.81379321
IGHM	862.4175	9.75224264
CTSH	755.695	9.56166027
TREM2	747.2575	9.54546166
ITGB2	723.2775	9.49840546
RGS10	706.555	9.46465806
SLCO2B1	677.38	9.40382158
ALOX5AP	606.6725	9.24477411
TNFAIP2	536.9825	9.06873126
P2RY12	526.465	9.04019381
S100A9	474.6525	8.89072787
OLFML3	441.1	8.78496195
CALR	435.9675	8.76807678
ITGAX	422.0725	8.72134702
ISG15	389.355	8.60494234
CD37	385.3125	8.58988518
CYBB	383.24	8.58210434
SPI1	348.0175	8.44301604
FCGR2A	291.7375	8.18852703
NCF4	284.915	8.15438777
FUS	275.245	8.10457255
CXCL10	272.61	8.09069467
GAS6	259.35	8.01875656
OLR1	259.15	8.01764358
LAIR1	256.285	8.00160523
CD163	255.3425	7.99628987
TSPO	245.8775	7.94179591

GPR34	234.75	7.87498135
HEXB	233.1925	7.86537758
MX1	232.575	7.86155222
MERTK	228.3775	7.83527671
TMEM173	227.92	7.83238372
CASP4	212.845	7.73365939
CCL2	201.3475	7.65354375
GAL3ST4	195.725	7.61268423
IL18	194.38	7.60273598
BHLHE41	193.9175	7.59929919
SLC2A5	193.68	7.59753117
IRF8	182.91	7.51499014
CPVL	179.2625	7.48592991
HCK	176.9875	7.46750366
ITGAL	171.3725	7.42099181
ANXA11	168.15	7.39360497
P2RY13	161.0875	7.33170074
ITGAM	154.3525	7.27008504
PILRA	150.885	7.23730558
TMEM119	148.245	7.21183964
BLNK	142.8825	7.15868542
TNFRSF1B	135.825	7.08560524
HAVCR2	134.61	7.07264178
PTAFR	134.5925	7.07245421
FPR1	133.11	7.05647515
ATP6V0A1	132.21	7.04668749
GBP2	131.925	7.04357417
TGFBR1	131.4275	7.03812337
ACP5	131.0275	7.03372583
SLC11A1	129.4425	7.01616757
SOD1	128.7525	7.00845664
EBI3	128.75	7.00842862
PTGS1	127.04	6.98913901
PTOV1	125.205	6.96814837
TLR2	117.9125	6.88157286
PTPRC	116.335	6.86214139
PFKFB3	112.3975	6.81246614
MMP14	111.02	6.79467579
CCND1	108.9075	6.7669595
DOK3	106.0925	6.72917886
NFKBIA	99.81	6.64111246
IL10RA	98.44	6.62117275
RAB3IL1	98.055	6.61551929
SSBP1	97.6925	6.6101759
ITGB5	96.5875	6.59376459

SLA	94.455	6.56155526
CD86	85.8425	6.42362019
PFDN1	84.91	6.40786257
IL1B	84.065	6.39343336
PTGES2	82.8225	6.37195085
BCL2L1	80.74	6.33521168
ENTPD1	80.0475	6.32278444
CD40	79.7175	6.31682456
CD33	77.4675	6.27551928
MX2	77.445	6.27510019
IFIT1	76.23	6.25228697
TIMP1	76.0125	6.24816478
PIK3AP1	75.8725	6.24550517
CFB	75.225	6.2331403
GBP3	75.205	6.23275668
ETV5	74.4575	6.21834527
VCP	73.4475	6.19864148
GBP5	72.7575	6.18502407
VPS13C	68.7675	6.10365499
PPARD	65.8475	6.04105676
ACSL1	65.5675	6.03490898
IFIT3	63.87	5.99706655
IGFBP4	60.8375	5.92688896
PLAUR	60.29	5.91384682
PPFIBP2	59.7	5.89965903
CEBPB	59.1525	5.88636724
PABPN1	57.535	5.84636795
PTPN7	57.0725	5.83472386
SLAMF7	55.8325	5.80303325
DKC1	55.4	5.79181407
PLAU	54.55	5.76950729
AGTRAP	54.4375	5.76652891
TLR1	53.77	5.74872957
ABCC5	52.8425	5.72362682
MSRA	52.185	5.70556327
GPR84	50.4775	5.65756855
TARDBP	49.1525	5.61919289
POLA2	49.1175	5.61816523
CAMK1	48.56	5.60169652
AGPAT1	48.325	5.59469783
SPN	47.8275	5.57976848
RCBTB2	47.1175	5.55819109
SLC7A8	46.82	5.54905303
RILPL2	46.1825	5.52927437
ENPP2	45.835	5.51837777

PIM1	45.4575	5.50644644
APBB3	45.0175	5.49241404
EML1	44.26	5.46793155
NLRP3	43.3325	5.43737757
IVNS1ABP	41.5575	5.37703696
CCR5	41.1875	5.36413466
FZR1	39.9925	5.32165756
MTSS1	39.745	5.31270147
RUNX3	39.235	5.2940693
PSTPIP2	38.9475	5.28345882
RASGRP3	38.945	5.28336621
RSAD2	36.235	5.17931199
SLC31A2	35.5	5.14974712
SESN1	35.4625	5.14822234
PROCR	35.3175	5.14231132
ANG	35.2025	5.13760598
ARHGEF7	35.2025	5.13760598
IFIT2	34.7825	5.12028973
CCL8	33.2775	5.05647515
NPEPPS	32.49	5.02192384
MMP9	32.125	5.00562455
ABCA7	31.3925	4.97234802
LAG3	31.29	4.96762975
APP	31.0925	4.95849472
UBE2E2	30.8825	4.94871764
SLC37A2	30.54	4.93262816
IL10	28.63	4.83945577
DNAJC9	28.5775	4.83680781
MCM3	28.325	4.82400405
HK3	28.2775	4.82158267
RUNX1	27.4	4.77610399
ARHGAP18	27.065	4.75835648
BATF	27.0175	4.75582228
CRYBB1	26.7625	4.74214099
RAPGEF2	25.5175	4.67341509
IGF1	25.4775	4.67115181
UPP1	25.0775	4.64832163
F13A1	24.7325	4.62833617
JAK2	24.6875	4.62570884
ZDHHC14	24.3075	4.60332962
GPD2	24.2825	4.60184506
GADD45B	23.7875	4.57213175
AGER	23.7425	4.56939995
ADRB2	22.8375	4.51333282
CD69	22.815	4.51191075

TNF	22.81	4.51159454
GCH1	22.705	4.50493813
MCM4	22.3825	4.48429928
CCRL2	22.1975	4.4723253
SUOX	21.75	4.4429435
PDGFC	21.71	4.44028782
CCL20	21.035	4.39471991
CD274	20.58	4.36317108
SPINT1	20.3675	4.348197
CREM	20.2	4.33628339
CD180	19.4075	4.27854238
PROS1	18.995	4.24754781
TRAF1	18.885	4.23916888
GYS1	17.935	4.16470584
PMEPA1	17.57	4.13504229
AKAP10	17.45	4.12515513
SMAD7	17.3775	4.11914864
SNAPC2	17.1475	4.09992635
F11R	17.045	4.09127669
NAMPT	16.79	4.06953033
IL1RN	16.7425	4.06544306
PRIM1	16.6475	4.05723364
NFKBIZ	15.5275	3.95675366
PHYH	15.455	3.95000175
LRRK2	15.4125	3.94602899
MCM6	14.7175	3.87946072
FAM102B	14.24	3.83187724
CABLES1	13.75	3.78135971
PDE4B	13.515	3.75648961
ARAP2	13.425	3.74685018
RBL1	13.075	3.70873904
CASP9	13	3.70043972
IL1A	12.9025	3.68957873
SLC6A12	12.57	3.65191274
BIRC3	12.54	3.64846544
EPAS1	12.215	3.61058196
IRAK3	11.9725	3.58165253
GK	11.94	3.57773093
RGS2	11.855	3.56742376
CABLES2	11.5975	3.53574194
CSF1	11.1675	3.48123435
CYSLTR1	10.9875	3.45779126
RAB11FIP1	10.675	3.41616416
MALT1	10.6125	3.40769265
FGD4	10.28	3.36176836

SDC2	10.15	3.34340782
NFRKB	9.51	3.24944534
DUSP16	9.3425	3.22380866
FN1	9.28	3.21412481
FCGR2B	9.12	3.18903382
GCNT1	8.1525	3.02724254
FLNB	7.89	2.9800253
SLC36A1	7.7825	2.96023367
TMEM154	7.285	2.86492897
CD80	6.7825	2.76181714
LAMP3	6.5525	2.71204545
TGM2	5.7175	2.51538446
HELLS	5.54	2.46988598
TET2	4.82	2.26903315
CD300E	4.64	2.21412481
CXCL8/IL8	4.4525	2.15461561
ENC1	4.3725	2.12845838
ZBED4	4.09	2.03210084
RMI1	3.7975	1.92504996
BNIP3	3.4675	1.79389588
MSH2	3.3725	1.75381844
CCR2	3.285	1.71589337
WDHD1	2.8275	1.49952702
POLA1	2.8025	1.48671437
ZC3H12C	2.62	1.38956681
XYLT1	2.5	1.32192809
IFT57	2.3225	1.2156786
SOCS3	2.1875	1.12928302
GOLM1	2.0575	1.04089243
IRF4	1.765	0.81966818
EXT1	1.675	0.7441611
DCBLD2	1.55	0.63226822
DKK1	1.4425	0.52857132
HGF	1.415	0.50080205
HGF/IL6	1.415	0.50080205
OPHN1	0.87	-0.2009127
ABCD2	0.5925	-0.7551129
SLC7A11	0.4425	-1.1762506
DGKH	0.4375	-1.1926451

Supplemental table 3. HIV induced transcriptional changes evaluated at 8 months of age (hg19)- Upregulation.

Gene	Expression levels, TPM		Fold change	Log2 fold change	p-value
	HIV+	Uninfected			
FXYD6	54.8975	3.405	16.1226138	4.011013748	0.01722811
C5orf42	40.5775	2.6725	15.18334892	3.92441813	0.03719037
CD300E	53.1875	4.64	11.46282328	3.518890516	0.02153112
SLC12A8	19.825	2.11	9.395734597	3.232005962	0.04076518
MCM3AP-AS1	32.53	3.6325	8.955264969	3.162736119	0.02019113
GALK2	37.57	5.4175	6.934933087	2.793881963	0.0272533
PTER	17.4325	2.6575	6.559736595	2.713637885	0.00134467
AC129507.1	44.8825	6.935	6.471881759	2.69418525	0.0017951
HELZ2	99.1625	16.305	6.081723398	2.604480203	8.44E-05
AC006027.1	68.2025	11.475	5.943572985	2.571330469	0.04293953
ACTA2	20.54	3.545	5.794076164	2.534578649	0.0038944
USP18	68.395	11.8975	5.748686699	2.523232407	0.00101022
IFIT1	431.19	76.23	5.656434475	2.499892939	7.98E-05
CDKN1A	167.785	29.93	5.605913799	2.48694956	0.04659772
CNTNAP2	11.515	2.125	5.418823529	2.437979665	0.00061455
TTC7B	77.52	14.69	5.277059224	2.399734175	0.02682365
HECTD4	34.5175	6.915	4.991684743	2.319526822	0.00530285
AL391058.1	117.1275	24.01	4.878279883	2.286372533	0.04205697
IFI6	1823.0875	379.2325	4.807308182	2.265229293	0.00017223
XIST	349.7375	73.0525	4.787481606	2.259266944	0.04912241
IFIT3	304.185	63.87	4.762564584	2.251738657	0.0002568
TXNDC5	102.7075	21.635	4.747284493	2.247102509	0.04562334
ZNF683	38.4625	8.345	4.609047334	2.204468584	0.00932802
GMPR	60.68	13.295	4.56412185	2.190337308	0.03146741
NEDD4L	40.755	9.0575	4.499585978	2.16979226	0.041285
MBTPS2	9.5375	2.1625	4.410404624	2.140911019	0.00117558
IFITM1	526.96	120.435	4.375472246	2.129438736	0.02522596
AC015911.7	28.085	6.435	4.364413364	2.125787749	0.01623068
ASGR2	33.8225	7.8	4.336217949	2.116437272	0.01680561
MBNL2	6.525	1.5125	4.314049587	2.109042759	0.01595465
TOP3B	45.4925	10.655	4.269591741	2.094098126	0.00108553
HIST1H2BN	152.0225	36.095	4.211732927	2.074413956	0.02474996
ISG15	1599.2325	389.355	4.107389144	2.038221638	0.0008069
RNA5-8SP6	8015.5375	1966.185	4.076695479	2.027400194	0.00380585
JPX	121.735	30.275	4.020974401	2.007545152	0.00958542
PSMD14	27.26	6.82	3.997067449	1.998941918	0.03796164
IFIT2	137.42	34.7825	3.950837346	1.982158453	0.00157318

IFNLR1	21.2125	5.4325	3.904739991	1.965226485	0.03687949
AC012651.1	16.6	4.2575	3.899001762	1.963104807	0.00096346
LY6E	1062.0225	272.7225	3.894150648	1.961308697	0.00035838
HSH2D	50.7625	13.07	3.883894415	1.957503981	0.02081094
AC093673.1	33.305	8.6625	3.844733045	1.94288343	0.01337471
PPP5C	66.9975	17.46	3.837199313	1.940053704	0.02622614
IFIT5	35.9425	9.4	3.823670213	1.934958098	0.02096718
MAPK12	29.0875	7.63	3.81225426	1.930644343	0.00697285
CABYR	31.535	8.2925	3.802833886	1.927074921	0.04899787
SNHG7	48.7875	12.9125	3.778315586	1.917743209	0.01511909
IFI44L	461.0425	124.3725	3.706948883	1.890232221	0.03882028
CMPK2	115.725	31.35	3.69138756	1.884163214	0.00015617
TMEM201	14.0775	3.8975	3.611930725	1.852770223	0.0452237
VAMP1	65.03	18.0575	3.601273709	1.848507253	0.03248122
ISG20	122.015	34.2575	3.561701817	1.83256674	0.02770207
C11orf21	34.77	9.775	3.557033248	1.83067446	0.02043909
RAB5B	32.905	9.2575	3.554415339	1.829612272	0.01606382
OAS3	151.3825	43.1925	3.504833015	1.80934571	0.00504472
ARMC6	59.6675	17.04	3.50161385	1.808019995	0.04161771
AC005786.3	82.79	23.93	3.459674049	1.790636122	0.03545831
OXR1	22.4075	6.54	3.426223242	1.776619156	0.0336437
IFITM3	1689.7025	496.675	3.402028489	1.766395223	0.00340145
GNL2	125.695	37.16	3.382534984	1.758104855	0.02038613
ZNF585B	12.4275	3.6775	3.379333787	1.756738857	0.01821105
COG4	127.4875	37.875	3.366006601	1.751038006	0.00480858
UBR5	34.325	10.36	3.313223938	1.728235717	0.03517541
SPP1	11336.925	3423.1525	3.311837553	1.72763191	0.02422009
OAS2	199.0725	60.9475	3.266294762	1.707654991	0.0043873
BAG6	258.8875	80.215	3.227420059	1.690381361	0.01688595
SCCPDH	18.05	5.63	3.206039076	1.68079201	0.0317956
NARS2	41.665	13.1525	3.167838814	1.66349893	0.03820782
GSPT2	6.0225	1.9125	3.149019608	1.654902741	0.02022108
VMO1	125.2875	40.02	3.130622189	1.646449411	0.02560827
RSAD2	111.875	36.235	3.087484476	1.626431883	8.09E-05
HAUS8	42.4625	13.8025	3.076435428	1.621259712	0.00143259
MX2	235.9325	77.445	3.046452321	1.607130161	0.01177996
PHKA2	49.7325	16.405	3.031545261	1.600053362	0.00079965
RAB11FIP1P1	10.9675	3.6625	2.994539249	1.582334042	0.03401157
STARD5	39.2025	13.0975	2.99312846	1.581654198	0.01887342
TRIM5	60.28	20.355	2.961434537	1.566296195	0.04046784
NFATC3	9.385	3.175	2.955905512	1.563600153	0.03903481
SMARCC1	27.2975	9.385	2.908630794	1.54034018	0.04051186

AEN	29.255	10.09	2.899405352	1.535757044	0.03299761
TNFSF14	20.45	7.0775	2.889438361	1.530789094	0.03593852
KIF17	19.83	6.8825	2.881220487	1.526680068	0.03257602
SPHK1	51.08	17.73	2.880992668	1.526565989	0.01386022
HPCAL1	42.2275	14.8225	2.848878394	1.510394041	0.00916086
AP002982.1	921.76	323.5625	2.848785011	1.51034675	0.01606452
UCKL1	45.7775	16.2125	2.823592907	1.497532102	0.01901915
PLBD2	19.3025	6.8725	2.808657694	1.489880806	0.00195576
FAM98C	39.7175	14.215	2.794055575	1.482360717	0.03020293
IFIH1	41.54	15.0075	2.767949359	1.468817548	0.04521309
MX1	641.22	232.575	2.757046114	1.463123398	0.00376143
HERC6	48.565	17.735	2.738370454	1.453317631	0.01909818
SIGLEC1	369.6875	135.2125	2.734122215	1.451077733	0.00438387
CES2	70.165	25.8675	2.712477046	1.439610929	0.03977778
AC010422.3	114.2575	42.735	2.673628174	1.418798841	0.01825083
HASPIN	6.75	2.525	2.673267327	1.418604114	0.04105267
EVA1B	45.9675	17.385	2.644089733	1.402771138	0.025539
MR1	61.5275	23.2875	2.642082662	1.401675605	0.02421024
ETV7	26.5325	10.1	2.626980198	1.393405326	0.01883436
RPAP1	37.925	14.4725	2.620487131	1.389835024	0.04736428
TIMP1	197.84	76.0125	2.602729814	1.380025555	0.03483499
HDAC7	107.7325	41.4425	2.599565663	1.378270597	0.02332255
SELL	65.355	25.325	2.58065153	1.367735345	0.03120468
NRBP1	41.695	16.2	2.573765432	1.363880575	0.04472702
EBAG9	17.095	6.645	2.572610986	1.363233318	0.03196026
IL1RN	42.9925	16.7425	2.567866209	1.360570037	0.02392997
PRPSAP1	68.585	26.8125	2.557948718	1.354987341	0.004984
TCF12	57.97	22.74	2.549252419	1.350074231	0.00231872
BUD23	211.6975	83.31	2.541081503	1.345442649	0.02824351
COPS6	82.8075	32.69	2.533114102	1.340912064	0.02329319
SPATS2L	169.415	67.52	2.509108412	1.327174807	0.00642607
RAB11FIP3	34.545	13.8075	2.501901141	1.323024784	0.01397924
IFITM2	556.315	222.6975	2.498074743	1.320816643	0.01588696
ICK	7.1725	2.885	2.486135182	1.313904744	0.02618431
NEK7	16.24	6.5925	2.463405385	1.300654061	0.00078794
CAPN7	30.7675	12.4925	2.462877727	1.300345005	0.00901615
STX5	49.6675	20.19	2.460004953	1.29866122	0.04731535
EPHB2	25.2025	10.29	2.449222546	1.292323869	0.038581
OAS1	446.7925	182.5675	2.447272926	1.291175004	0.01220056
GMPPB	22.71	9.32	2.436695279	1.284925846	0.02466833
PPWD1	53.115	21.97	2.417614929	1.273584475	0.04998702
MICU1	40.26	16.7075	2.409696244	1.268851298	0.02869661

IFI35	235.875	97.9725	2.407563347	1.267573758	0.02775028
RNF34	94.6875	39.45	2.400190114	1.263148683	0.00643375
IFI44	674.8575	284.085	2.375547811	1.248260243	0.00540845
VILL	40.895	17.4125	2.348600144	1.231801111	0.04817763
HCST	526.885	225.1175	2.340488856	1.226809896	0.04017428
COASY	100.41	42.98	2.336202885	1.224165569	0.00789228
SHISA5	306.3425	131.225	2.334482759	1.223102934	0.00857355
LGALS3BP	821.935	353.0775	2.327916676	1.21903942	0.00107446
NLRC5	211.815	91.185	2.322914953	1.215936335	0.03574026
HDLBP	266.6225	114.78	2.322900331	1.215927254	0.01867685
REEP5	80.445	34.685	2.319302292	1.21369087	0.0104106
IL34	5235.9625	2260.5875	2.316195458	1.211757004	0.01385128
HNRNPA3P1	107.37	46.845	2.292026897	1.196623974	0.01912132
SFXN3	96.195	41.9975	2.290493482	1.195658457	0.00030336
PLGRKT	47.6475	20.8675	2.28333533	1.191142749	0.00774333
RRP1	42.8425	18.7725	2.2821947	1.190421877	0.04019875
QRICH1	114.4775	50.2525	2.278045868	1.187796796	0.0102212
SUOX	49.505	21.75	2.276091954	1.186558844	0.04536761
SMAP1	21.9775	9.675	2.271576227	1.183693719	0.00731976
NR1H2	91.3125	40.3325	2.263993058	1.178869534	0.00035857
IL1B	189.505	84.065	2.254267531	1.172658741	0.02812462
RTP4	42.27	18.9675	2.228548834	1.156104575	0.0412145
MGLL	30.35	13.7725	2.203666727	1.139906054	0.0398221
RNFT1	46.4475	21.1825	2.192729848	1.132728078	0.01148532
ACADM	79.7775	36.395	2.191990658	1.13224165	0.01595399
CH25H	85.5175	39.0425	2.190369469	1.131174243	0.03769408
TOR1B	45.1125	20.63	2.186742608	1.128783417	0.03108384
CACTIN	30.5275	13.965	2.186000716	1.128293874	0.03974614
MTPAP	8.92	4.1075	2.171637249	1.118783135	0.02360391
SDHA	118.5875	54.685	2.168556277	1.116734884	0.00577409
ANXA6	84.6625	39.165	2.161687731	1.112158132	0.0398193
WDR5	30.9	14.3275	2.156691677	1.108819942	0.02337343
MBOAT1	9.8575	4.5875	2.148773842	1.103513647	0.02648039
APOBEC3G	104.9625	48.925	2.145375575	1.101230232	0.00200234
CD151	96.75	45.16	2.14238264	1.099216175	0.028464
NMI	70.14	32.86	2.134510043	1.09390495	0.0140442
TNFRSF10B	101.275	47.6775	2.124167584	1.086897591	0.0089161
SERPINH1	43.1825	20.5275	2.103641457	1.072888833	0.00693881
SYAP1	16.52	7.8675	2.099777566	1.070236508	0.01266433
FYCO1	18.54	8.895	2.084317032	1.059574733	0.04269229
SETD5	38.3425	18.505	2.072007566	1.051029271	0.03549894
TOLLIP	67.2775	32.5625	2.066103647	1.046912629	0.03275751
MRPL35	51.1325	24.815	2.060548056	1.04302811	0.02171999

MAN2A2	123.61	60.46	2.044492226	1.031742577	0.03344275
NFU1	82.7475	40.4775	2.044283861	1.031595537	0.0483747
PARP10	292.6075	143.94	2.032843546	1.023499185	0.00704
SECTM1	57.6775	28.405	2.030540398	1.02186373	0.04551786
ZNF326	25.25	12.46	2.026484751	1.01897932	0.01497348
HSPA9	133.155	65.8075	2.023401588	1.016782682	0.04221055
PML	129.9375	64.2525	2.022294852	1.015993359	0.00728337
DYNLT1	244.61	121.2375	2.017610063	1.012647376	0.01484999
TMCO1	90.8975	45.0875	2.016024397	1.011513098	0.00178524
SLTM	183.5175	91.0375	2.015845119	1.011384798	0.03624916
HERC5	43.0525	21.4275	2.009217128	1.006633478	0.0244648
TINF2	126.5625	63.0525	2.007255858	1.005224524	0.0378379
CCDC127	2.895	1.4425	2.006932409	1.004992029	0.03921369
PARP12	210.2	104.8	2.005725191	1.004123952	0.00055625
ATP1B1	41.7925	20.9675	1.993203768	0.995089206	0.02860235
RNASEH2A	43.145	21.6725	1.990771715	0.993327795	0.04461548
MBOAT7	68.0975	34.455	1.976418517	0.982888477	0.04095627
RIPK3	78.79	40.31	1.954601836	0.966874752	0.00574341
KIAA0319L	51.955	26.585	1.954297536	0.96665013	0.01259776
CCR1	166.94	85.5425	1.951544554	0.9646164	0.00078514
PAAF1	66.9575	34.52	1.939672654	0.955813198	0.01450768
MPPE1	158.6175	82	1.934359756	0.951856135	0.02232493
PLOD3	69.8725	36.535	1.912481182	0.935445553	0.04664106
ZFR	22.1225	11.595	1.907934455	0.93201161	0.00242315
TRIM21	119.3625	62.6475	1.905303484	0.930020814	0.03865759
DCAF8	139.415	73.295	1.90210792	0.927599103	0.03311806
THOP1	73.72	38.975	1.89146889	0.919507048	0.01046026
GALK1	90.655	47.9475	1.890713802	0.918930998	0.02842298
SLC25A19	131.48	69.955	1.87949396	0.910344279	0.04240096
METTL9	66.02	35.1475	1.878369728	0.909481063	0.02729067
GDE1	71.465	38.1	1.875721785	0.907445857	0.00347739
VRK3	52.37	28.0475	1.867189589	0.900868422	0.04650228
TMEM189	33.095	17.735	1.866084015	0.900013941	0.00254348
RBM17	149.9475	80.67	1.858776497	0.894353308	0.01047087
STRADA	87.34	47	1.858297872	0.893981774	0.03602651
LRP3	52	28.1925	1.84446218	0.883200207	0.04244487
SLC27A3	88.58	48.2	1.837759336	0.877947851	0.03497788
CNOT7	41.8825	22.8025	1.836750356	0.877155554	0.01697915
ANKRD13A	68.89	37.875	1.818877888	0.863048689	0.01262843
TRAPPC12	155.92	86.3725	1.805204203	0.852162042	0.01127491
XPNPEP1	99.2	54.9725	1.804538633	0.85163003	0.04882624
SERF2	169.68	94.38	1.797838525	0.846263449	0.01420876
TBC1D23	14.125	8.0025	1.765073415	0.819728191	0.04312028

CCDC90B	48.61	27.6325	1.759160409	0.814887041	0.03070393
FDPS	122.055	69.4975	1.756250225	0.81249841	0.04435234
NT5C3B	29.1075	16.685	1.744531016	0.802839247	0.03299313
NCF1	281.9	162.9075	1.730429845	0.791130453	0.04140804
MALT1	18.345	10.6125	1.728621908	0.789622351	0.01777768
AL583722.2	138.0625	80.0375	1.724972669	0.786573504	0.00980444
SAMD9	29.0025	16.8325	1.723006089	0.7849278	0.02430492
MYD88	111.9425	64.99	1.722457301	0.78446822	0.00466745
APOE	8114.485	4766.6875	1.70233207	0.767512488	0.00223839
SERPING1	324.76	193.4375	1.67888853	0.747506445	0.02403163
PEPD	160.485	95.925	1.673025801	0.742459695	0.02451472
SLC39A13	69.275	41.775	1.65828845	0.729694977	0.0238834
AP2S1	233.1625	140.725	1.656866228	0.728457127	0.04021659
ARFRP1	54.7825	33.21	1.64957844	0.722097382	0.03192148
NBEAL2	130.5025	79.19	1.647966915	0.720687279	0.02653422
TEX261	60.4475	36.7725	1.643823509	0.717055411	0.01296086
MLF2	177.3	108.0425	1.641020895	0.714593608	0.00663493
CPSF2	30.0375	18.475	1.625845737	0.701190379	0.02911171
BANF1	169.285	104.295	1.623136296	0.698784149	0.00933547
TMC6	220.38	135.975	1.620739106	0.696651876	0.03024743
CPSF7	73.4075	45.7325	1.605149511	0.682707683	0.04974627
BCL10	7.635	4.805	1.588969823	0.668091726	0.04955636
ABHD3	82.85	52.3775	1.581786072	0.661554497	0.02077089
RTL8C	106.6775	67.6625	1.57661186	0.656827533	0.01388373
SELENOI	5.125	3.255	1.574500768	0.654894461	0.0469405
RRP1B	18.125	11.5175	1.573692208	0.654153397	0.04888736
PRDX4	62.6875	40.1375	1.561818748	0.643227036	0.03098512
DNAJB6	76.6175	49.115	1.559961315	0.641510253	0.0347495
PQBP1	145.665	93.92	1.550947615	0.633149959	0.00485876
IARS	39.855	25.7175	1.549722951	0.632010324	0.01646515
LAP3	330.2	214.465	1.539645164	0.622597897	0.01472875
UBE2L6	354.62	230.495	1.538514935	0.621538449	0.00364238
PLD3	627.9825	411.175	1.527287651	0.610971807	0.01484993
DTX3L	62.14	40.7975	1.523132545	0.607041492	0.01433418
RNPEPL1	166.815	109.8025	1.519227704	0.603338119	0.01388568
ACADVL	706.93	472.335	1.496670795	0.581756924	0.01478106
FAM53B	44.97	30.1025	1.493895856	0.579079577	0.00962204
APOL2	85.9625	57.6875	1.490140845	0.575448698	0.04406798
NAP1L4	123.185	84.4375	1.458889711	0.544870823	0.0257546
CAPZA1	145.2925	99.8975	1.454415776	0.540439754	0.04255287
CDK5RAP3	579.575	400.3375	1.44771599	0.533778605	0.01094975
PSMD6	66.09	46.29	1.427738172	0.513731433	0.01527164
LTA4H	58.365	41.355	1.411316649	0.497041713	0.02796928

RETREG2	72.77	52.345	1.390199637	0.475292073	0.04323823
PHRF1	24.44	17.7	1.38079096	0.465494925	0.01513471
RBM5	141.56	103.3975	1.369085326	0.453212363	0.03174175
EIF3B	89.1825	66.03	1.350636075	0.433638996	0.04969659
SEC13	127.2825	95.6975	1.330050419	0.411480936	0.03735926
BCAP31	256.7825	194.7575	1.318472973	0.398867998	0.00145672
PSME1	309.7375	235.5525	1.314940406	0.394997417	0.04674935
PKM	518.7575	397.525	1.304968241	0.384014696	0.04718986
GSN	393.395	309.14	1.272546419	0.347718283	0.0252138
CREG1	414.175	327.3825	1.265110383	0.339263268	0.00436985
LGMN	467.27	396.0875	1.179714078	0.238437243	0.02048691

Supplemental table 4 HIV induced transcriptional changes evaluated at 8 months of age (hg19)- Downregulation.

Gene	Expression levels, TPM		Fold change	Log2 fold change	p-value
	HIV+	Uninfected			
PSAP	2051.445	2395.52	0.856367302	-0.223698383	0.04075024
PFDN5	670.95	809.8325	0.82850466	-0.271418283	0.00714326
FCER1G	1611.695	1971.515	0.81749061	-0.290725935	0.0332362
NANS	78.3275	97.7475	0.801324842	-0.319540892	0.04997244
RPS2	1271.8975	1601.1025	0.794388554	-0.332083259	0.03186983
RPS3	587.6775	741.025	0.793060288	-0.334497552	0.04685608
NCF4	225.88	284.915	0.792797852	-0.334975041	0.01165333
MRFAP1	179.115	228.4925	0.783898815	-0.35126065	0.04243293
LHFPL2	100.9	129.4675	0.779346168	-0.359663812	0.00697366
GPX4	429.0225	551.4675	0.777965157	-0.362222553	0.03947836
RACK1	687.215	884.2625	0.777161759	-0.363713181	0.04615489
CD276	130.9375	169.015	0.774709345	-0.368272952	0.04857593
ACTB	3507.84	4611.2675	0.760710586	-0.394580414	0.00269018
TGFBR1	98.995	131.4275	0.753228967	-0.408839613	0.00583488
ITGB2	542.995	723.2775	0.750742281	-0.413610359	0.01801761
AKR1A1	404.055	541.81	0.745750355	-0.423235335	0.04698739
SSR2	169.12	226.9575	0.745161539	-0.424374882	0.00757413
OS9	311.3325	421.0575	0.739406138	-0.435561075	0.03064039
IL13RA1	117.3175	160.46	0.731132369	-0.451795469	0.03783597
PA2G4	37.65	51.5075	0.73096151	-0.452132653	0.04413321
RPLP1	1079.5	1497.9	0.720675613	-0.472578069	0.03302154
RPS29	2506.52	3484.2925	0.719377033	-0.475179995	0.03706166
ATF4	186.9375	260.5075	0.717589705	-0.478768903	0.00620799
HLA-DMA	772.2125	1082.985	0.713040809	-0.487943448	0.00789469
CD74	19736.6575	27696.205	0.712612342	-0.488810625	0.01795829
RPS10	563.38	797.2425	0.706660771	-0.500910272	0.0129883
TMEM176B	313.2175	445.18	0.703574958	-0.507223959	0.04367624
RPL23A	830.13	1186.9775	0.699364563	-0.5158834	0.00351166
LRRFIP1	49.2475	70.4525	0.699017068	-0.516600412	0.04928587
RPL36A	301.6025	432.335	0.697612962	-0.519501248	0.0429623
SH3PXD2A	4.8975	7.0225	0.69740121	-0.519939227	0.0225899
IKZF1	37.9575	54.4625	0.696947441	-0.520878233	0.03924119
ZW10	7.655	10.9875	0.696700796	-0.521388883	0.0248265
SERPINB6	198.98	285.7525	0.696336865	-0.522142691	0.00693775
MFSD12	125.13	179.7275	0.696220667	-0.522383454	0.03647708
RASSF1	75.645	109.095	0.693386498	-0.528268351	0.01651714

MT-ND2	3838.21	5542.125	0.692552045	-0.530005603	0.00298653
RPS20	965.145	1400.245	0.689268664	-0.536861667	0.00219552
PBX2	44.6975	65.04	0.687230935	-0.541133116	0.02060735
MT-CO1	8192.345	11927.96	0.686818618	-0.541998948	7.46E-05
EPB41L2	156.81	228.33	0.68676915	-0.542102861	0.04528479
WASF2	53.43	77.9225	0.685681286	-0.544389947	0.04578531
RPL41	2564.2575	3761.4	0.681729542	-0.552728592	0.00537304
HMGN2	266.7475	391.4875	0.681369137	-0.553491494	0.04102571
RPSA	574.0875	843.195	0.680847847	-0.554595669	0.00613432
STARD7	58.985	86.7175	0.680197192	-0.555975045	0.04022418
GRINA	173.935	256.7825	0.677363138	-0.561998617	0.04818836
RPLP2	816.3425	1206.0275	0.676885477	-0.563016331	0.00056498
MT-ND1	3181.665	4710.6075	0.675425622	-0.566131187	0.00143875
RPS18	830.52	1239.7975	0.66988359	-0.578017684	0.01949816
COTL1	633.8375	946.705	0.669519544	-0.578801925	0.00022156
RASSF2	41.29	61.6825	0.669395696	-0.579068821	0.03586731
CELF2	15.825	23.8075	0.6647065	-0.589210633	0.00967623
LPIN2	35.995	54.155	0.664666236	-0.589298026	0.03751514
ELOVL5	30.3175	45.6225	0.664529563	-0.589594712	0.04846253
EIF3H	86.16	129.795	0.663816018	-0.591144654	0.04058342
MT-ND5	1723.11	2597.125	0.663468258	-0.591900649	0.01151547
RPL13A	1647.2325	2483.615	0.663239874	-0.59239735	0.00778706
RUNX3	26.01	39.235	0.662928508	-0.593074801	0.02206209
ATXN7L3B	18.025	27.2225	0.662136101	-0.594800305	0.0401715
RPS8	659.5125	997.5675	0.661120676	-0.59701446	0.00572345
RPLP0	626.7125	948.3975	0.660812054	-0.597688091	0.00409875
SLC30A1	9.5125	14.41	0.660131853	-0.599173882	0.01855688
NACA	781.59	1184.1525	0.660041675	-0.599370975	0.00306942
CYBRD1	14.3025	21.73	0.65819144	-0.60342083	0.04272551
RPL18	594.295	903.2725	0.657935451	-0.603982044	0.00986875
COPA	35.985	54.76	0.657140248	-0.605726788	0.0476734
SPATA20	120.02	183.11	0.655453006	-0.609435747	0.02257996
RNF166	163.145	250.4425	0.651426974	-0.618324635	0.03197327
RPL8	863.745	1326.7075	0.651044032	-0.619172975	0.0134967
MT-ATP6	7143.215	10975.81	0.650814382	-0.619681963	0.00152545
ZC3H3	9.8325	15.1925	0.647194339	-0.627729106	0.04783406
SRSF3	36.2825	56.1025	0.646718061	-0.628791194	0.0196329
TTC31	31.4625	48.6775	0.646345848	-0.629621764	0.03738286
EIF3F	136.585	211.37	0.646189147	-0.629971575	0.04268818
RPL12	678.74	1053.1425	0.644490181	-0.633769717	0.03267661
SAR1B	9.4575	14.7	0.643367347	-0.636285379	0.03576995
RPS16	871.4125	1359.8975	0.64079278	-0.642070202	0.0024311

RPL6	773.16	1208.2425	0.639904655	-0.644071134	0.00795042
RPL26	1717.0225	2690.3675	0.638211137	-0.647894311	0.00120641
NFATC2	17.6625	27.7225	0.637117865	-0.650367804	0.01092985
SLC25A6	203.4725	319.5	0.636846635	-0.650982109	0.00422793
CD46	47.3375	74.455	0.635786717	-0.65338522	0.01000988
MT-CO2	7045.5975	11092.525	0.635166249	-0.65479384	0.00158884
RPS7	365.52	576.1525	0.634415367	-0.656500377	0.0335527
RPL37A	829.9125	1312.0275	0.632542001	-0.660766817	0.01100166
PHC2	74.205	117.465	0.631720087	-0.662642648	0.00015435
MFSD1	146.635	232.185	0.631543812	-0.663045273	0.04511541
RPL27A	1018.37	1614.6525	0.630705368	-0.664961882	0.01307422
PRDX3	89.9475	142.615	0.630701539	-0.664970641	0.01322307
MANF	63.35	100.4725	0.630520789	-0.665384156	0.03042545
RPL21	1176.86	1868.9875	0.629677834	-0.667314214	0.00188829
RPL18A	636.7525	1012.2275	0.629060661	-0.668728951	0.00302099
C1orf162	212.6525	338.0825	0.628995881	-0.668877525	0.02082969
KLHL36	12.33	19.6175	0.628520454	-0.6699684	0.01756588
RPS11	1939.635	3100.1675	0.625654904	-0.676560973	0.00130214
FAU	427.8225	685.95	0.623693418	-0.681091061	0.03009518
MT-ND4L	4423.185	7120.5025	0.621190007	-0.686893473	0.00128006
AKAP17A	53.84	86.695	0.621027741	-0.687270381	0.02920783
RPL10A	408.4275	658.745	0.620008501	-0.689640098	0.02142761
TGOLN2	53.595	86.4725	0.61979242	-0.690142985	0.03655799
PTP4A2	34.25	55.4025	0.61820315	-0.69384709	0.03903203
COL8A2	15.4525	25.075	0.616251246	-0.698409436	0.02947421
MDH2	72.1125	117.02	0.616240814	-0.69843386	0.0008415
ATL3	8.805	14.335	0.614230903	-0.703146995	0.03002856
PYCARD	190.1225	309.64	0.614011433	-0.703662577	0.04779365
RPS23	1169.2025	1906.545	0.613257227	-0.705435764	0.0110706
RPL13	1406.7625	2294.485	0.613105991	-0.705791593	0.00717492
P4HA1	11.9	19.4225	0.612691466	-0.706767338	0.04422268
RPS12	508.4875	833.215	0.610271659	-0.712476503	0.01140883
MTATP6P1	736.0575	1206.9025	0.609873208	-0.713418755	0.00910884
TKT	94.09	154.4175	0.60932213	-0.714722956	0.00996391
RPS25	558.5925	918.5375	0.608132493	-0.717542418	0.02496528
LST1	491.975	811.1	0.606552829	-0.721294788	0.04791303
ZBTB38	4.3375	7.155	0.606219427	-0.722088009	0.04479529
MS4A6A	673.2725	1115.565	0.603526016	-0.728512133	0.00609357
C6orf48	131.3925	217.7875	0.603305975	-0.729038225	0.04401576
CSF2RB	20.78	34.4525	0.603149263	-0.72941302	0.0443603
RPS24	1650.9475	2746.4525	0.60111999	-0.734275097	0.00210261
TBRG1	39.89	66.7725	0.597401625	-0.743226933	0.00884004

HLA-DMB	678.9775	1141.875	0.594616311	-0.749969057	0.00171658
FYB1	51.335	86.65	0.592440854	-0.755256964	0.01948229
YWHAQ	30.895	52.2075	0.591773213	-0.756883702	0.03983854
CORO7	117.375	198.9175	0.590068747	-0.761045047	0.0154039
UBE3A	5.62	9.5425	0.588944197	-0.763797151	0.0407029
CERKL	15.9225	27.0575	0.588469001	-0.764961675	0.02302372
PSMG2	47.19	80.435	0.586684901	-0.769342232	0.01965195
ATP5F1	135.8425	231.6425	0.586431678	-0.769965057	0.02469478
RPL5	227.25	390.3825	0.582121381	-0.780608087	0.02247044
ANP32B	83.69	143.95	0.581382424	-0.782440636	0.02452811
RPL7A	702.2425	1209.4325	0.580638027	-0.784289036	0.00075261
MT-ND4	4721.1075	8135.99	0.580274496	-0.785192572	0.00135799
RPL10	1579.08	2724.815	0.579518243	-0.787074019	0.00123491
HAUS4	54.84	95.15	0.576353127	-0.794975085	0.01713806
RPS13	682.625	1184.845	0.576130211	-0.795533183	0.02360328
ATP5I	262.365	455.795	0.575620619	-0.796809824	0.03544664
BTAF1	24.05	41.8225	0.575049316	-0.79824241	0.01487377
RPL30	192.01	334.3825	0.574222634	-0.800317896	0.01832763
LITAF	121.425	211.5825	0.573889618	-0.801154821	0.04358381
PAXX	99.4475	173.9125	0.571824912	-0.806354622	0.00658694
EIF3C	203.26	357.4	0.568718523	-0.814213303	0.04856546
DNAJC5	43.0325	75.71	0.568385946	-0.815057211	0.03151467
RPL23	492.9825	869.1325	0.567212134	-0.8180397	0.00164015
APMAP	62.0425	109.8375	0.564857175	-0.824041968	0.00889463
BHLHE41	109.295	193.9175	0.563615971	-0.827215602	0.00975059
MT-ND3	4613.8275	8202.19	0.562511658	-0.830045097	0.00067486
KIAA0141	92.925	165.4075	0.561794356	-0.831885963	0.03736427
SEC16A	44.8	79.7725	0.561597042	-0.832392759	0.00108079
KLHL5	21.75	38.805	0.560494782	-0.835227154	0.0014683
IRF5	132.695	236.76	0.560462071	-0.835311351	0.02700433
VPS29	123.8825	221.2275	0.559977851	-0.83655833	0.01961567
CCDC88A	16.595	29.6925	0.558895344	-0.839349939	0.03488553
SPAG7	34.425	61.7275	0.557693087	-0.842456708	0.02339008
SETX	33.3475	60.165	0.554267431	-0.851345858	0.04566122
SNHG12	70.4725	127.485	0.552790524	-0.855195209	0.01524386
APOL3	56.7275	103.1225	0.550098184	-0.862238954	0.02690578
ELOF1	23.655	43.025	0.54979663	-0.863030031	0.03918347
UBAC1	44.285	80.6675	0.548981932	-0.865169427	0.02162475
HLA-DOA	111.2775	202.895	0.548448705	-0.8665714	0.00547023
METTL17	90.4925	165.015	0.54838954	-0.866727041	0.04935837
RPL4	695.02	1267.5925	0.548299237	-0.86696463	0.02999126
RPL11	544.455	995.7075	0.546802148	-0.870909184	0.00228628
TMEM167A	23.2475	42.755	0.543737575	-0.879017567	0.02115864

RPS28	393.2425	723.2725	0.543698952	-0.879120049	0.03693214
TPT1	3860.32	7115.8525	0.542495787	-0.882316162	1.10E-06
HLA-DRB1	3243.2225	5978.9075	0.542444	-0.882453888	0.02469749
RPS9	667.195	1230.5775	0.542180399	-0.883155137	0.00304167
SEC11A	99.1475	183.225	0.541124301	-0.885968064	0.03882605
NUCKS1	16.0575	29.6925	0.54079313	-0.886851272	0.045224
MT-CO3	5552.0425	10279.1025	0.540129111	-0.888623787	0.00014052
CD93	30.51	56.505	0.539952217	-0.889096354	0.01134515
HLA-DRA	6031.9725	11190.0775	0.539046535	-0.891518272	0.00537775
EEF1A1	1291.7075	2403.25	0.537483616	-0.895707317	0.00012428
DRAM2	61.01	113.5425	0.537331836	-0.896114777	0.03644861
REX1BD	116.365	216.6075	0.537215932	-0.896426004	0.03221315
RPL7	606.945	1132.3475	0.536005952	-0.899679073	0.00021332
RPL3	852.7325	1591.24	0.53589182	-0.8999863	0.00239998
RPS6	733.7225	1369.3325	0.535824937	-0.900166371	2.91E-05
RPL22	306.1275	573.665	0.533634613	-0.906075849	0.00876244
AC004656.1	8.08	15.1525	0.533245339	-0.907128644	0.03465173
RPL27	447.7525	841.375	0.532167583	-0.910047464	0.00392557
ADGRG5	14.685	27.84	0.527478448	-0.922815946	0.02686851
ZWINT	18.525	35.33	0.524341919	-0.931420207	0.03763294
MT-ND6	2735.58	5225.5025	0.523505634	-0.933723031	0.00038184
SLC9A3R1	46.0025	87.875	0.523499289	-0.933740518	0.02460085
DDT	51.3725	98.355	0.522317117	-0.937002113	0.00822523
LAMTOR1	93.7	179.8025	0.521127348	-0.940292127	0.02260386
NDUFB1	181.315	349.675	0.518524344	-0.947516372	0.04585752
HLA-DQB1	1262.27	2465.4725	0.511978941	-0.965843625	0.02005358
RPL37	463.9375	906.415	0.511837845	-0.966241272	0.00074797
SLC2A5	99.0175	193.68	0.511242772	-0.967919555	0.00755285
LAMTOR5	50.0175	98.335	0.508643921	-0.975272051	0.04613596
HLA-DPB1	1931.8275	3811.3675	0.506859415	-0.980342446	0.00302821
MBD6	20.285	40.0875	0.506018085	-0.982739146	0.01971744
KRI1	27.9125	55.205	0.505615433	-0.983887594	0.04188474
SACM1L	18.1675	36.17	0.502280896	-0.993433692	0.03188857
NAGPA	23.305	46.8875	0.497040789	-1.008563845	0.01562029
AMACR	21.045	42.35	0.496930342	-1.00888446	0.04311884
SLC7A8	23.23	46.82	0.496155489	-1.01113578	0.02059552
MEFV	5.2925	10.675	0.495784543	-1.012214801	0.04140583
ZNF589	14.72	29.7025	0.49558118	-1.012806693	0.02190804
RPL34	421.835	851.6675	0.495304799	-1.013611495	0.00023694
MSL1	24.3325	49.7575	0.489021756	-1.032029446	0.00546639
ANKZF1	39.22	81.37	0.481995822	-1.052907455	0.0277992
SCML1	9.71	20.18	0.481169475	-1.055382974	0.04574849
ECSIT	19.465	40.56	0.479906312	-1.059175308	0.0455406

EPM2AIP1	3.99	8.3875	0.475707899	-1.071852115	0.0421454
KRCC1	20.21	42.61	0.474301807	-1.07612273	0.01547251
EEF1G	393.745	831.4225	0.473579919	-1.078320187	0.00043153
SIRPB2	27.29	57.6375	0.473476469	-1.078635366	0.03319723
COX15	17.4875	37.09	0.471488272	-1.08470621	0.03149857
CCDC84	40.2475	85.8875	0.46860719	-1.093549006	0.0182045
ZFAND2A	17.5725	37.5775	0.467633557	-1.096549634	0.04118077
RPS26	432.7725	932.71	0.463994704	-1.107819757	0.03100607
EBI3	59.735	128.75	0.463961165	-1.107924042	0.01010638
ZC3H4	8.37	18.0675	0.463262765	-1.110097366	0.04219684
BIN1	327.8025	712.31	0.460196403	-1.119678387	0.01061013
ZDHHC6	46.7525	101.635	0.460003936	-1.12028189	0.03579945
ZNF512B	6.415	14.0675	0.456015639	-1.132844793	0.04152823
E4F1	12.205	26.7775	0.45579311	-1.133548979	0.03936372
ARL16	20.7	45.455	0.455395446	-1.13480823	0.02213471
NNT	22.1825	48.8	0.454559426	-1.137459179	0.03813349
SYF2	36.6225	80.5975	0.454387543	-1.138004811	0.04216241
PRAM1	56.25	123.8825	0.45405929	-1.139047401	0.04550551
NAP1L1	91.7025	202.4075	0.453058805	-1.142229778	0.02264612
IL12RB1	23.0225	50.8975	0.452330665	-1.144550292	0.0286918
RPS3A	491.81	1087.745	0.45213722	-1.145167411	0.00037295
RPL35A	224.01	496.23	0.451423735	-1.14744582	0.00977769
CDK19	6.575	14.6375	0.449188728	-1.154606371	0.03679414
C5AR2	11.3325	25.275	0.448367953	-1.157244931	0.03941235
RPS27A	367.97	825.0825	0.445979644	-1.164950231	0.00012699
GUSBP11	35.93	81.4175	0.441305616	-1.180149988	0.0419878
NUBP2	34.3475	77.87	0.44108771	-1.180862531	0.04593083
NUSAP1	16.5725	37.575	0.441051231	-1.180981851	0.00196785
VPS52	38.87	88.275	0.440328519	-1.183347808	0.00674394
ZNF224	24.7825	56.545	0.438279247	-1.190077729	0.04959686
JMJD1C	19.1275	43.7875	0.436825578	-1.19487076	0.02626707
RNF187	85.1	194.8775	0.436684584	-1.195336494	0.02464489
GMEB2	3.6625	8.4225	0.434847136	-1.201419764	0.04781202
TFEC	25.385	58.47	0.434154267	-1.203720331	0.04069318
CLDND1	30.3225	69.9125	0.433720722	-1.205161721	0.02890583
FDX1	6.6425	15.3275	0.433371391	-1.206324177	0.03244448
POLG	37.95	87.615	0.433145009	-1.207078	0.03983547
LSM7	60.9725	141.245	0.431678997	-1.211969192	0.0496124
NABP1	55.95	130.8325	0.427646036	-1.225510928	0.00412258
MT-CYB	2042.5775	4793.1575	0.426144457	-1.230585528	7.39E-05
PCBP1-AS1	16.2425	38.1525	0.425725706	-1.23200389	0.03641419
SMAD4	16.3	38.5025	0.423349133	-1.24008016	0.01256025
ZNF18	12.5075	29.5725	0.422943613	-1.241462759	0.02570307

MTND2P28	54.7125	129.395	0.422833185	-1.241839487	0.00450842
RPL31	389.195	920.6725	0.422729038	-1.242194879	0.00217877
CXCL9	28.215	67.2075	0.419819217	-1.25215989	0.02618355
RPL9	331.57	797.015	0.416014755	-1.265293397	9.76E-05
TCAF1	4.5175	11.005	0.410495229	-1.284562639	0.03682035
HIRIP3	8.0775	19.7275	0.409453808	-1.288227389	0.02964328
RPL24	153.5275	377.1225	0.407102467	-1.296536132	0.04636483
ILVBL	21.875	53.7525	0.406957816	-1.297048838	0.04441566
DCP2	11.9475	29.39	0.406515822	-1.298616591	0.04941693
CASP4	86.0125	212.845	0.404108624	-1.307184956	0.00977747
ZNF736	3.43	8.49	0.404004711	-1.307555977	0.04325665
CTBP1-AS2	6.2	15.4375	0.401619433	-1.316099016	0.00853592
DUSP12	11.3675	28.3125	0.401501104	-1.316524141	0.01624553
RRP36	23.1775	58.015	0.399508748	-1.323701002	0.00389919
MRPL53	63.6825	159.835	0.398426502	-1.32761448	0.01562935
EEF1A1P5	27.8475	69.9025	0.39837631	-1.327796237	0.01911647
8-Mar	5.015	12.6	0.398015873	-1.329102128	0.04464444
VNN1	2.835	7.135	0.397337071	-1.331564695	0.01987798
DEAF1	21.1775	53.3475	0.396972679	-1.332888375	0.01342542
MED1	4.5575	11.5075	0.396046057	-1.336259881	0.00676781
TOMM22	16.22	40.99	0.39570627	-1.33749817	0.03148414
LRRC75A-AS1	136.515	345.155	0.395517956	-1.338184903	0.00435596
HLA-DRB5	1213.0225	3070.2825	0.39508498	-1.339765095	0.01466187
GNPTAB	11.8975	30.2975	0.392689166	-1.3485403	0.00170073
EAF1	3.52	8.99	0.391546162	-1.352745687	0.0280705
VARS	36.0925	93.23	0.38713397	-1.36909519	0.01515095
SLC46A1	12.1475	31.4375	0.38640159	-1.371827062	0.04368972
CCS	43.2075	111.9925	0.385807085	-1.374048456	0.01126395
TTL	11.2625	29.1925	0.385801148	-1.37407066	0.0364527
RAB12	24.1575	62.8075	0.384627632	-1.378465684	0.03630921
ALG3	22.6975	59.255	0.383047844	-1.384403494	0.04384799
NDUFAF3	46.535	121.58	0.382752097	-1.385517812	0.01036286
PHYH	5.8975	15.455	0.381591718	-1.389898236	0.04722963
GLA	43.0825	113.185	0.380637894	-1.393508901	0.04812377
TRO	19.02	50.2525	0.378488632	-1.401678124	0.02688522
SIRT1	5.8625	15.5725	0.376464922	-1.40941265	0.00637475
NAA25	4.225	11.25	0.375555556	-1.412901755	0.02469053
DISP1	9.265	24.685	0.375329147	-1.413771764	0.01989988
PHF10	5.9075	15.94	0.370608532	-1.432032	0.01826552
NOL8	19.3775	52.385	0.369905507	-1.434771315	0.00194472
ZNF304	3.5925	9.7375	0.368934531	-1.438563266	0.0108217
EIF3L	156.61	426.4075	0.36727778	-1.445056478	0.01536216
FEM1B	5.0475	13.81	0.365496017	-1.452072409	0.01695678

PIAS3	8.175	22.5425	0.362648331	-1.463356886	0.01969118
MRE11	4.9225	13.6025	0.361882007	-1.466408717	0.04901018
WDR24	6.84	19.11	0.357927786	-1.482259548	0.016745
CD209	10.92	30.545	0.35750532	-1.483963384	0.01954888
PCGF3	24.835	69.6225	0.356709397	-1.487178872	0.01490372
ZNF75D	5.935	16.83	0.352644088	-1.503715242	0.02703161
HIPK3	3.9375	11.2525	0.34992224	-1.514893736	0.02453928
ZNF211	10.29	29.41	0.349880993	-1.515063802	0.03467689
IFT80	10.02	28.6425	0.349829798	-1.515274912	0.00343472
VPS13A	5.7525	16.585	0.346849563	-1.527618029	0.03166918
FAM89B	18.57	53.77	0.345359866	-1.533827656	0.01840948
OPRL1	6.1525	18.09	0.34010503	-1.55594775	0.00925951
MFF	34.085	100.68	0.338547874	-1.562568233	0.0157299
ADAMDEC1	46.5675	137.9825	0.33748845	-1.567089967	0.00615383
HLA-DQA1	645.895	1916.17	0.337076042	-1.568854004	0.00666447
KDM3A	9.9475	29.5425	0.336718287	-1.570386019	0.02642738
TBC1D8	8.6675	25.825	0.335624395	-1.575080513	0.0340004
KCNMB1	3.87	11.575	0.334341253	-1.580606722	0.01126607
VPS37B	7.9425	23.9325	0.331870887	-1.591306019	0.00400105
AKAP1	9.265	28.1425	0.329217376	-1.602887613	0.01043133
MSI2	3.5975	10.95	0.328538813	-1.605864278	0.02814228
AL135999.1	7.0425	21.4475	0.328359949	-1.606649928	0.03481437
UHRF2	8.02	24.4475	0.328049903	-1.608012801	0.02042237
RAPGEF2	8.3425	25.5175	0.326932497	-1.612935306	0.03316493
UBN1	12.3875	37.9525	0.326394836	-1.615309864	0.03195514
TPM1	11.8825	36.495	0.325592547	-1.618860419	0.04569269
VPS25	10.94	33.83	0.323381614	-1.628690439	0.02018335
CD5	6.8675	21.375	0.32128655	-1.63806751	0.0378127
LIN7C	2.355	7.4825	0.31473438	-1.667793314	0.03882907
ZFP14	1.86	5.91	0.314720812	-1.667855509	0.0484938
GART	12.96	41.5175	0.312157524	-1.679653855	0.00943632
PATZ1	7.59	24.895	0.304880498	-1.713684225	0.02507117
RDH10	1.0825	3.5525	0.304714989	-1.714467625	0.03598957
AC108488.1	5.46	17.95	0.304178273	-1.717010988	0.03979518
TLR8	1.9575	6.4475	0.303606049	-1.71972756	0.03110817
PDPR	6.745	22.24	0.303282374	-1.72126644	0.00878122
GPANK1	7.5625	25.04	0.302016773	-1.72729942	0.03193143
SLC35F6	6.825	22.69	0.300793301	-1.733155658	0.03575491
PWAR6	0.9225	3.0725	0.300244101	-1.735792194	0.01444984
SLAMF8	32.185	109.015	0.295234601	-1.760066283	0.0108246
ADAT1	8.4275	28.55	0.295183888	-1.760314118	0.01387202
KIAA0556	4.165	14.185	0.293620021	-1.76797775	0.04045875
SUPT3H	4.28	14.74	0.29036635	-1.784053823	0.04979595

PLCB3	12.2725	42.6875	0.28749634	-1.798384507	0.03695294
DENND4C	4.56	15.995	0.28508909	-1.810515263	0.02684437
TRPV4	4.295	15.1075	0.28429588	-1.814534906	0.02244899
ASL	13.67	48.47	0.282030122	-1.82607884	0.03795254
RAE1	6.63	23.51	0.282007656	-1.826193764	0.00182836
PRPF39	4.6925	16.7	0.280988024	-1.831419453	0.02695933
SNIP1	2.3575	8.41	0.280321046	-1.83484803	0.00084664
NDRG2	44.44	159.0825	0.279351909	-1.839844418	0.00112143
UBA5	10.8275	38.76	0.279347265	-1.839868398	0.03311162
PPIAP22	9.93	35.7325	0.277898272	-1.847371232	0.03360568
IL7R	3.1475	11.3825	0.276520975	-1.854539176	0.04626773
ABCA11P	3.525	12.8175	0.275014628	-1.862419735	0.04673053
POGLUT1	4.9075	17.9025	0.274123726	-1.867100894	0.01137295
GPALPP1	4.38	16.0375	0.273109899	-1.87244649	0.0155184
SPATA7	3.98	14.6625	0.27144075	-1.881290772	0.02955662
SAP130	2.5225	9.3375	0.270147256	-1.888182069	0.04200161
ZNF226	10.085	37.35	0.270013387	-1.888897159	0.01496245
ARMCX5	3.515	13.1425	0.26745292	-1.90264314	0.0160017
RNF138	5.7575	21.7025	0.265292017	-1.914346831	0.03838238
SYT17	7.595	28.64	0.265188547	-1.914909623	0.02309671
PMPCB	12.4375	47.155	0.26375782	-1.922714227	0.00839446
KLF9	1.25	4.78	0.261506276	-1.935082523	0.01681469
FAM13A	11.3425	44.6875	0.253818182	-1.978132677	0.02511744
ZBTB14	4.8475	19.2625	0.25165477	-1.990482153	0.03346694
QSOX2	2.8075	11.175	0.251230425	-1.992916904	0.03012847
SCMH1	4.8325	19.4375	0.248617363	-2.008001038	0.01168111
FCHO2	6.8225	27.445	0.248588085	-2.008170945	0.0468389
AFDN	6.515	26.2825	0.247883573	-2.012265428	0.02881652
ZNF236	2.925	11.805	0.247776366	-2.012889512	0.021364
FAM84B	1.885	7.6475	0.24648578	-2.020423678	0.03723314
MLLT10	4.075	16.795	0.242631736	-2.043159831	0.03026458
DDX12P	2.755	11.3925	0.241825763	-2.047960147	0.01313628
PGBD2	2.545	10.8025	0.235593613	-2.08562767	0.01213717
FLVCR2	7.8825	33.7	0.233902077	-2.096023421	0.03146415
ANKRD36B	2.7	11.56	0.233564014	-2.098110085	0.00606973
ZNF331	4.74	20.4925	0.231304136	-2.112137034	0.03155124
AK1	21.4325	92.71	0.231177866	-2.112924818	0.02986502
GAREM1	1.21	5.3	0.228301887	-2.130985312	0.01477019
PQLC2	7.74	34.315	0.225557336	-2.148433883	0.02432552
TTC39C	6.9225	30.83	0.224537788	-2.154969836	0.02894402
RPL9P9	22.7525	101.7875	0.223529412	-2.161463423	0.0197795
RAB3D	1.2175	5.56	0.21897482	-2.191163111	0.03127605
CNTLN	1.1725	5.355	0.218954248	-2.191298652	0.02306869

AAMDC	9.5575	43.6825	0.218794712	-2.192350226	0.01482666
AL732372.3	6.985	32.425	0.2154202	-2.214774554	0.02193308
EXD2	3.065	14.245	0.215163215	-2.216496643	0.04858144
DLG1	4	18.6425	0.214563497	-2.220523436	0.03423528
EPC2	2.395	11.175	0.214317673	-2.22217727	0.04272618
MYL6B	15.7625	74.6925	0.211031897	-2.244467016	0.02114829
SAP30L	11.525	54.885	0.209984513	-2.251645166	0.03587317
RNLS	3.825	18.3925	0.207965203	-2.265585938	0.00081261
AL162586.1	6.6325	32.255	0.205627035	-2.281898141	0.03261863
GFER	9.15	44.6525	0.204915738	-2.286897303	0.04484157
ENOSF1	18.8075	92.575	0.2031596	-2.299314554	0.00593047
FMO5	2.2425	11.2	0.200223214	-2.320318842	0.04103743
PIWIL4	4.2025	21.0325	0.199809818	-2.32330062	0.0100289
ZNF100	1.34	6.8625	0.195264117	-2.356501243	0.02298093
UXT	14.955	76.8325	0.194644194	-2.361088787	0.01414663
TMEM223	4.0825	21.0775	0.193689954	-2.368178968	0.02731805
UNC119B	2.4925	12.8825	0.193479526	-2.369747183	0.02609711
CLP1	2.7875	14.77	0.18872715	-2.405626116	0.0010319
ZNF7	7.815	41.7725	0.187084805	-2.41823571	0.0011651
PPFIA4	2.805	15.0525	0.186347783	-2.423930441	0.04359453
ELMOD2	3.855	20.785	0.185470291	-2.430739983	0.01370101
GON7	6.355	34.41	0.184684685	-2.436863862	0.04350399
KLHL26	1.355	7.35	0.184353741	-2.439451398	0.04252214
SF3A2	16.21	93.72	0.172962015	-2.531472863	0.00664108
C19orf25	7.07	41.89	0.168775364	-2.566823764	0.0227355
ZNF528	4.1975	25.0725	0.167414498	-2.578503625	0.04642038
KCTD21	1.1975	7.2775	0.164548265	-2.603417278	0.03395731
LDHD	4.625	29.445	0.157072508	-2.670497403	0.0289778
UPK3BL1	19.555	127.3725	0.153526075	-2.703444389	0.0068206
CSNK1A1	5.1825	34.185	0.15160158	-2.721643309	0.01392632
IL10	4.185	28.63	0.146175341	-2.774228142	0.01159677
ISCA1	5.7775	40.605	0.142285433	-2.813140128	0.0017808
PDE6B	3.435	24.4075	0.14073543	-2.828942527	0.01163976
RHOBTB1	3.005	21.7075	0.138431418	-2.852756688	0.0047713
PHF6	0.9625	6.9675	0.138141371	-2.855782652	0.00286195
ZNF71	1.5525	11.5125	0.13485342	-2.890535983	0.01463618
ZNF223	1.8175	14.2475	0.12756624	-2.970681523	0.01642124
RABL2A	2.5425	20.105	0.126461079	-2.983234657	0.04339063
TFAP4	2.2775	22.7925	0.09992322	-3.323036216	0.001432
HLA-DRB6	11.33	116.3875	0.097347224	-3.360716355	3.58E-05
DNLZ	3.125	35.825	0.087229588	-3.51903861	0.00491926
CHRFAM7A	1.955	24.1775	0.080860304	-3.628424562	0.03625006
MTA3	1.1525	14.495	0.079510176	-3.652716678	0.00788376

IL1RAP	0.9475	12.58	0.075317965	-3.730862169	0.01423708
SCAPER	0.955	14.0875	0.067790594	-3.882771067	0.01225635
HDAC9	1.9975	41.19	0.04849478	-4.366026718	0.01406397
LINC00909	1.2425	40.49	0.030686589	-5.026247886	0.00697624
SH3BP5	0.1525	8.06	0.018920596	-5.723898691	0.03634056
